# Phosphorylation Stoichiometries of Human Eukaryotic Initiation Factors

**DOI:** 10.3390/ijms150711523

**Published:** 2014-06-27

**Authors:** Armann Andaya, Nancy Villa, Weitao Jia, Christopher S. Fraser, Julie A. Leary

**Affiliations:** Department of Molecular and Cellular Biology, University of California at Davis, Davis, CA 95616, USA; E-Mails: aaandaya@ucdavis.edu (A.A.); nvilla@ucdavis.edu (N.V.); wtjia@ucdavis.edu (W.J.); csfraser@ucdavis.edu (C.S.F.)

**Keywords:** mass spectrometry, eukaryotic initiation factor, translation, phosphorylation quantification

## Abstract

Eukaryotic translation initiation factors are the principal molecular effectors regulating the process converting nucleic acid to functional protein. Commonly referred to as eIFs (eukaryotic initiation factors), this suite of proteins is comprised of at least 25 individual subunits that function in a coordinated, regulated, manner during mRNA translation. Multiple facets of eIF regulation have yet to be elucidated; however, many of the necessary protein factors are phosphorylated. Herein, we have isolated, identified and quantified phosphosites from eIF2, eIF3, and eIF4G generated from log phase grown HeLa cell lysates. Our investigation is the first study to globally quantify eIF phosphosites and illustrates differences in abundance of phosphorylation between the residues of each factor. Thus, identification of those phosphosites that exhibit either high or low levels of phosphorylation under log phase growing conditions may aid researchers to concentrate their investigative efforts to specific phosphosites that potentially harbor important regulatory mechanisms germane to mRNA translation.

## 1. Introduction

The process of converting nucleic acid to functional protein, a process involving the precise spatial and temporal arrangement of proteins, protein complexes, and nucleic acids, is known as translation [1,2,3]. Initiation, elongation, termination, and recycling constitute the four chronological phases of mRNA translation with initiation bearing the greatest regulation. The principal molecules that regulate initiation for eukaryotes are the eukaryotic initiation factors (eIFs), a family which is comprised of 12 members encompassing at least 25 individual proteins [1]. One of the most common means of regulating proteins involves phosphorylation, a well-established post-translational modification. Herein, we report our global investigation of phosphorylation quantification for three essential eukaryotic initiation factors, eIF2, eIF3, and eIF4G.

At the onset of eukaryotic translation initiation, the 40S ribosome binds several factors including eIF2 and eIF3 to form the 43S preinitiation complex (PIC). eIF2 (a heterotrimer consisting of the proteins eIF2α, eIF2β, and eIF2γ) binds as a ternary complex (TC) comprised of eIF2, the initiator methionyl tRNA (Met-tRNA_i_), and GTP (guanosine triphosphate). The PIC can then bind to nascent messenger RNA (mRNA) through the direct interaction of eIF3 and eIF4G, a member of the eIF4F cap-binding complex. The largest of the eIFs, eIF3 is comprised of thirteen distinct subunits with a holoprotein mass of approximately 800 kDa. Numerous studies implicate the holoprotein eIF3 and its many subunits as an essential factor during translation initiation: It is the central assembly on which other factors, proteins, and nucleic acids bind [4,5,6,7,8,9,10]. The factor eIF4F is also an essential protein to translation initiation. It is comprised of three other eIFs: eIF4A, eIF4E, and the largest of the three, eIF4G with a mass of 176 kDa. eIF4G functions as a scaffolding protein onto which other factors, such as eIF3, eIF4A, and eIF4E functionally interact [11,12,13].

Although each subunit has a defined function during translation initiation, the regulatory mechanisms governing initiation have yet to be entirely solved. Protein phosphorylation, albeit a well-established post-translational modification, is still not completely understood, particularly in eIFs, as regards target specificity. However, the resulting effects once a protein is phosphorylated can be dramatic as that of ser-51 phosphorylation on eIF2α, which has been extensively studied and serves as a prime example of the effect phosphorylation has on the initiation process.

During initiation, eIF2 TC hydrolyzes GTP then releases Met-tRNA_i_ following start codon recognition. eIF2B, a GTP exchange factor (GEF) for eIF2, exchanges GDP (guanosine diphosphate) for GTP, which then promotes binding of a new Met-tRNA_i_ for further rounds of translation. Phosphorylation of eIF2α at ser-51 reduces the dissociation rate of eIF2 from eIF2B, effectively sequestering eIF2B and preventing eIF2 TC regeneration and thus globally repressing the rate of mRNA translation [14,15,16,17,18]. The largest protein of the three subunits, eIF2γ, functions as a scaffolding protein on which the remaining subunits bind. We recently identified eight novel sites of phosphorylation on eIF2γ and demonstrated the potential *in vitro* effects of eIF2γ phosphorylation via protein kinase C (PKC) [19].

In addition to the identification of novel phosphosites, determining those levels of phosphorylation on specific residues allows researchers to define potentially important phosphosites, thereby distinguishing those sites from potentially less biologically meaningful ones [20,21,22,23,24,25,26]. As with eIF2α, phosphorylation at ser-51 becomes more pronounced under conditions of cellular stress which demonstrates how the fluidity of the phosphorylation stoichiometry reveals intrinsic mechanisms implicated in regulating phosphorylation in response to cellular cues [14,15,16,17,18]. Establishment of phosphosite stoichiometry under specific biological conditions may help to focus ensuing investigations for deciphering biologically important phosphosites.

While identification of novel phosphosites is the essential first step in the eventual evaluation of their biological impact, follow-up studies, even on a few novel sites of phosphorylation, often presents a task too burdensome for subsequent in-depth investigations. Researchers identify phosphosites for further study through evaluation of their structural location. Phosphosites residing in structurally salient locations such as established binding sites, binding pockets, or areas of significant secondary, tertiary, and/or quaternary structure, are considered desirable candidates for further evaluation. However, without such informative data, further investigations into newly discovered phosphosites are often avoided.

In order to assess the amount of phosphorylation occurring at specific residues, we recently published a mass spectrometry method proficient at measuring phosphosite stoichiometry [27]. Our method relies on the measurement of dephosphorylation of phosphopeptides accomplished chemically via cerium oxide [28]. We have further optimized cerium oxide’s capacity for dephosphorylating peptides and have subsequently developed a method to measure the amount of dephosphorylation via tandem mass tags (TMT) [25]. Use of the isobaric TMT reporter ions allows for assessment of phosphorylation stoichiometry. We have verified the use of this quantification protocol and have shown its efficacy in measuring the stoichiometry of previously established phosphosites. One of the subunits of eIF3, eIF3h, has one previously identified phosphorylation site, ser-183 [29], and we have now determined its level of phosphorylation to be 70% in log phase grown HeLa cells (data provided and discussed in the results section). This observation coincides with the critical *in vivo* effects of eIF3h’s ser-183 phosphorylation during malignant transformation of NIH 3T3 cells [27,30].

This report is the first to highlight the phosphorylation levels of three heavily phosphorylated eukaryotic initiation factors. Additionally, our quantification analysis is specific to the analysis of cells grown at optimal conditions and thus underscores the importance of variability possible with these phosphosites under different environmental circumstances. Knowledge gained from this study provides a platform for future investigations not only for phosphorylated proteins, but also for the inherent variability of specific phosphosites within the protein itself. Hence, the purpose of this study is to add to the growing pool of knowledge of these factors and more importantly, to initiate an investigation primarily aimed at quantified phosphorylated residues and their implication on translation initiation. Future studies into the regulation of these factors may be based on the findings within this study.

## 2. Results and Discussion

### 2.1. Quantification of Phosphorylation for Eukaryotic Initiation Factor 2 (eIF2)

We isolated eIF2 from HeLa cell lysate in order to quantify its level of phosphorylation. The factor was purified from HeLa cells under optimal growth conditions (log phase growth). As eIF2 is a heterotrimer with a molecular mass of 126 kDa, we analyzed the two factors that have been previously reported as phosphorylated within HeLa cells, eIF2β and eIF2γ. While eIF2α has been heavily studied at its principally phosphorylated residue of ser-51, quantification of that residue does not lead to new information or developments, which is the principal aim of this study. Thus, we focused our efforts at quantifying the numerous sites on the remaining two subunits of eIF2 in order to gain further insight into their function and regulation.

**Figure 1:**
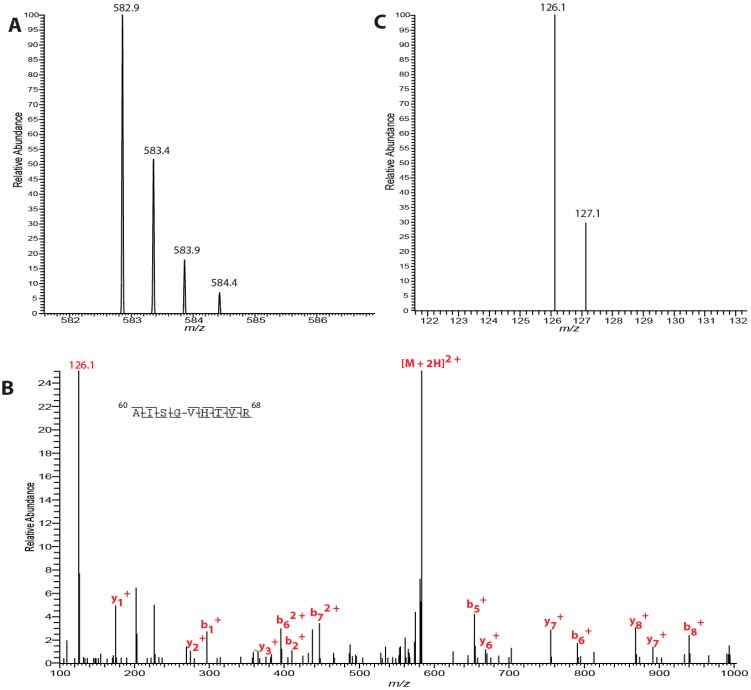
Quantification of phosphorylation on Thr-66 on eIF2γ. (**A**) Precursor ion mass scan of the [M + 2H]^2+^ ion is shown; (**B**) MS/MS spectra of *m*/*z* ion 582.9 illustrating indicative b- and y-ions for peptide spanning residues 60-68 of eIF2γ; and (**C**) Zoomed in view of the *m*/*z* region containing the TMT (tandem mass tags) reporter ions. Calculation of the reporter ion ratio reveals a phosphorylation level of 70.5% for Thr-66.

Recently, we published the identification of eight sites of phosphorylation, seven of which were novel and reside on the largest subunit, eIF2γ, the core subunit of the heterotrimer [19]. All identified phosphosites were quantified. On eIF2γ, numerous functional domains exist dedicated to specific tasks during eukaryotic translation. Threonine 66, a phosphorylated residue, is located within the switch-1 region of the protein, and in this study, we observed its level of phosphorylation at 71% (Figure 1). Two sites adjacent to one another, ser-55 and thr-56, reside directly in the nucleotide binding pocket of eIF2γ and our quantitative phosphoanalysis revealed the levels of phosphorylation to be 85% for both ser-55 and thr-56 combined (Supplementary Information). Proximal to the *C*-terminal end of the protein, ser-412 and thr-413 exhibit phosphorylation at 7% while ser-418 and thr-435 have phosphorylation at 70% and 60%, respectively (Supplementary Information). Lastly, thr-109’s phosphorylation level was observed at 30% and this residue sits adjacent to the zinc binding domain (Supplementary Information).

In contrast to eIF2γ, eIF2β has a lower abundance of phosphorylated residues within HeLa cells. Nevertheless, we quantified these sites and observed levels of 18% for thr-31 but near or less than 5% for the other reported eIF2β phosphosites (Table 1).

**Table 1:** Three of the factors, eIF2, eIF3, and eIF4G were analyzed as to their quantification of phosphorylation levels within log phase grown HeLa cells. Percentages of the phosphosites are shown.

Protein	Subunit	Residue Phosphorylated	% Phosphorylation
eIF2	β	Thr-31	18
		Ser-67	<5
		Ser-105	<5
		Thr-111	<5
		Ser-158	8
	γ	Ser-55	85
		Thr-56	85
		Thr-66	71
		Thr-109	30
		Ser-412	7
		Thr-413	7
		Ser-418	70
		Thr-435	66
eIF3	a	Ser-881	84
		Ser-1198	92
		Ser-1336	18
		Ser-1364	36
	b	Ser-83	85
		Ser-85	85
		Ser-119	70
		Ser-125	70
	c	Thr-524	95
	g	Thr-41	31
		Ser-42	31
	h	Ser-183	89
	j	Thr-109	88
eIF4G	-	Thr-647	65
		Ser-1028	5
		Ser-1077	15
		Ser-1092	40
		Ser-1144	45
		Ser-1147	45
		Ser-1185	70
		Ser-1187	70
		Ser-1209	50
		Thr-1211	50
		Ser-1231	75
		Thr-1425	62
		Ser-1430	62
		Ser-1596	<5

### 2.2. Quantification of Phosphorylation for Eukaryotic Initiation Factor 3 (eIF3)

Although the exact structure and placement of each subunit within the tridodecamer of eIF3 has yet to be fully solved, the number of phosphorylations derived from log phase HeLa cells is 29 [29]. We thus sought to quantify the levels of each of these 29 sites of phosphorylation. Given that each subunit is a distinct protein, the varying levels of phosphorylation from subunit to subunit or within each subunit should not be surprising.

**Figure 2:**
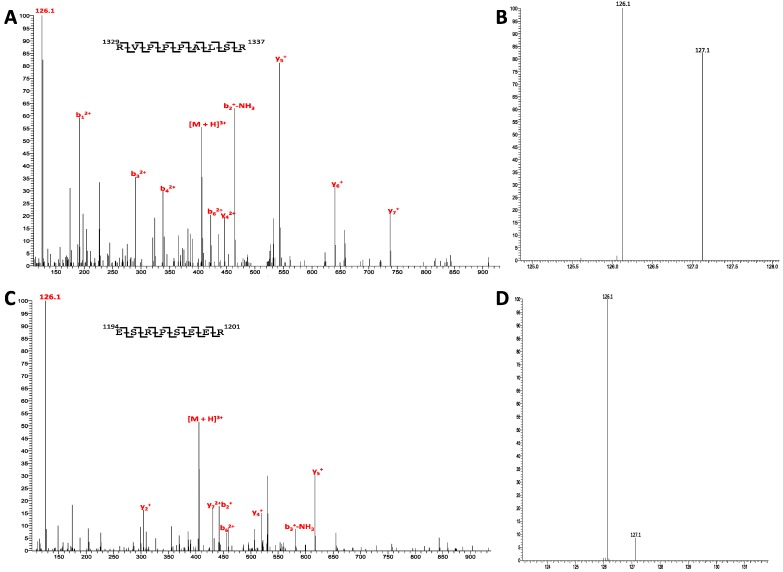
Quantification of phosphorylation on eIF3. (**A**) MS/MS spectra of *m*/*z* ion 406.6, *z* = 3. Peptide spans residues 1329–1337 for eIF3a; (**B**) Zoomed in view of the *m*/*z* region containing the TMT reporter ions. Calculation of the reporter ion ratio reveals a phosphorylation level of 18% for Ser-1336; (**C**) MS/MS spectra of *m*/*z* ion 405.5, *z* = 3. Peptide spans residues 1194–1201 for eIF3a; and (**D**) Zoomed in view of the *m*/*z* region containing the TMT reporter ions. Calculation of the reporter ion ratio reveals a phosphorylation level of 92% for Ser-1198.

The core subunits of eIF3 are widely believed to be eIF3a, eIF3b, and eIF3c, the three largest of the thirteen subunits. These three subunits house 21 of the 29 sites of phosphorylation for eIF3, 15 of which were accurately quantified. The largest subunit, eIF3a, has a varied landscape of phosphorylation ranging from 18% for ser-1336 to a high of 92% for ser-1198 (Figure 2). Intriguing are the two phosphorylated residues at the *C*-terminal end (or *C*-Terminus) of the protein, ser-1336 and ser-1364. While ser-1336 has a phosphorylation of 18%, ser-1364 has a level of 36% (Supplementary Information). The exact crystal structure of human eIF3a has yet to be solved, but from this study, we see that the *C*-terminal phosphorylated residues are lower in phosphorylation levels than that for ser-1198 at 92% and for that of ser-881 at 84%. This rather high level of phosphorylation for these residues may implicate functional significance for eIF3 quaternary structure given that the *C*-terminal phosphorylated residues possess a lower level of phosphorylation.

The second largest eIF3 subunit, eIF3b, has 7 sites of phosphorylation. Unfortunately, we did not attain full sequence coverage for all proteins analyzed which lead to a lack of quantification for some previously identified phosphosites. This was due in part to either the absence of a lysine or arginine within the vicinity of the phosphosite in question or an inadequacy of sufficient identifiable b- and y-ions. However, as with eIF3a’s ser-1198 and ser-881, the levels of phosphorylation for eIF3b were relatively large ranging from 70% for both ser-119 and ser-125, and 85% for both ser-83 and ser-85 (Supplementary Information). Again, implications in eIF3 quaternary structure for these high levels of phosphorylation will be investigated further.

The third largest purported eIF3 core subunit is eIF3c. Again, due to the same limitations as previously mentioned, the *N*-terminal phosphorylated residues of ser-9, ser-11, ser-13, ser-15, ser-16, ser-18, and ser-39 could not be quantified. But as with subunits eIF3a and eIF3b, higher levels of phosphorylation were observed for thr-524 at 95% (Supplementary Information).

Regarding the subunit eIF3h, we have already published a report detailing its relatively high level of phosphorylation from log phase HeLa cells at 70% and within the current study, 89% [27]. This high level of phosphorylation is in direct correlation with biological data showing that mutation of this residue to alanine produced a decrease in growth rate of NIH-3T3 cells [30]. Residues not quantified for eIF3 due to previously mentioned experimental limitations were eIF3f (ser-258) and the *N*-terminal residues of eIF3j (ser-11, ser-13, and ser-20). A level of 31% phosphorylation was observed for eIF3g’s two phosphorylated residues, thr-41 and ser-42, and a high level of phosphorylation comparable to that of eIF3h was observed for thr-109 on eIF3j (Table 1 and Supplementary Information).

### 2.3. Quantification of Phosphorylation for Eukaryotic Initiation Factor 4G (eIF4G)

A large scaffolding protein, eIF4G commonly occurs with varying isoforms that are dependent on the location of the start codon. Nonetheless, the principal isoform of the protein is comprised of 1599 amino acids with a molecular mass of 176 kDa. Prior to translation initiation, eIF4G serves as a docking site to which other proteins bind prior to forming larger complexes necessary for the translation process. Known proteins that bind to eIF4G are poly A binding protein (PABP), the kinase Mnk1, and initiation factors eIF4E, eIF4A, and eIF3. The phosphorylation of eIF4G from HeLa cells has been well documented with evidence of up to 20 sites of phosphorylation corresponding to eIF4G derived from HeLa cells have been identified. However, as with all other eIFs, the quantification of this phosphorylation has yet to be reported.

**Figure 3:**
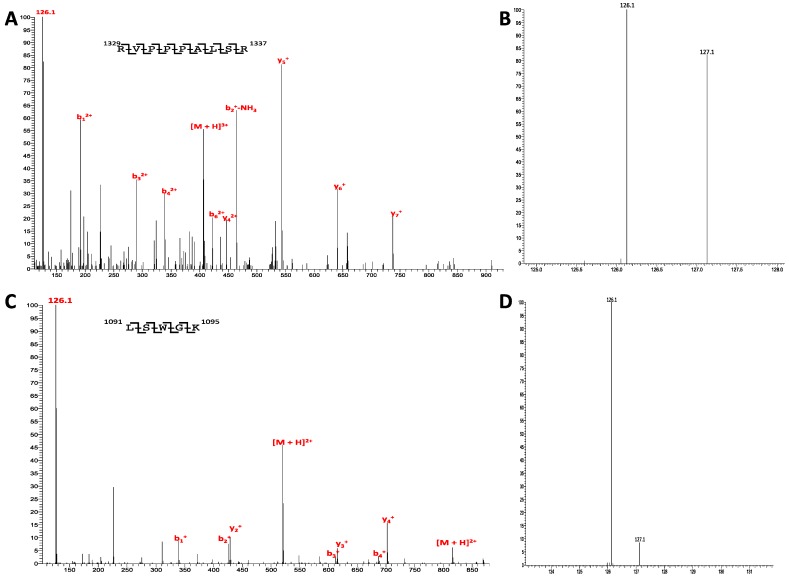
Quantification of phosphorylation on eIF4G. (**A**) MS/MS spectra of *m*/*z* ion 992.6, *z* = 2. Peptide spans residues 1072–1085; (**B**) Zoomed in view of the *m*/*z* region containing the TMT reporter ions. Calculation of the reporter ion ratio reveals a phosphorylation level of 15% for Ser-1077; (**C**) MS/MS spectra of *m*/*z* ion 520.8, *z* = 2. Peptide spans residues 1091–1095; and (**D**) Zoomed in view of the *m*/*z* region containing the TMT reporter ions. Calculation of the reporter ion ratio reveals a phosphorylation level of 40% for Ser-1092.

Currently, considerable investigation has focused on the portion of eIF4G that has been mapped to bind to eIF3 [31]. This portion, corresponding to residues 1015 to 1105, contains three sites of phosphorylation identified from HeLa cells, ser-1028, ser-1077, and ser-1092. Quantification of each site of phosphorylation reveals 5%, 15%, and 40% for ser-1028, ser-1077, and ser-1092, respectively (Figure 3). The only other site of phosphorylation that maps to a known binding region on eIF4G is located at the *C*-terminus of the protein and is ser-1596 that corresponds to the Mnk1 binding region. Quantification of its phosphorylation reveals less than 5% levels of phosphorylation.

As for the remaining known sites of phosphorylation for eIF4G from HeLa cells, insufficiently scored peptides prevented our quantitative analysis of phosphosites residing in the *N*-terminal portion of the protein. However, high levels of phosphorylation were observed for thr-647 at 65%. Regions near but distinct from the eIF3 binding site had phosphorylation levels at or near 50%. The peptide mapped to ser-1144 and ser-1147 had a combined level of 45% and the peptide mapped to ser-1209 and ser-1211 was at 50%. The peptide mapping to ser-1185 and ser-1187 had a level of 70% and the peptide mapping to ser-1231 had a level of 75%. Peptides mapping in close proximity to the Mnk1 binding region, thr-1425 and ser-1430 were quantified at phosphorylation levels of 62%.

The entire process of translation initiation requires a concerted and choreographed effort from numerous proteins to effectively translate the nascent mRNA into functional protein. Although the process of translation may be separated into the four distinct phases of initiation, elongation, termination, and recycling, much of the regulation is centralized to the initiation phase. Translation initiation is the process by which the 40S ribosome, bound to Met-tRNA_i_ and several initiation factors, binds to the 5' end of mRNA then scans in the 5' to 3' direction until the initiation codon is recognized. Recruitment of Met-tRNA_i_ requires formation of a ternary complex also containing eIF2 and GTP, while mRNA recruitment to the ribosome complex requires binding of eIF4G and eIF3.

As the formation of the ternary complex is critical to translation, a better understanding of the major protein component, eIF2, would help define this process. A closer look into the function of eIF2 reveals that the heterotrimer consists of three subunits, eIF2α and eIF2β bound to eIF2γ but not each other. Classically, eIF2α’s phosphorylation on ser-51 converts the eIF2 heterotrimer into an inhibitor of eIF2B, the heteropentamer responsible for exchanging GDP for GTP on eIF2. Since the phosphorylation of this one residue on eIF2α has a profound impact on the overall process of translation, we hypothesized that the other subunits of eIF2 may also contribute in some part to the overall process of translation.

Our phosphoanalysis of eIF2 centered on known phosphosites for both eIF2β and eIF2γ derived from HeLa cells. In our previous report for eIF2γ, one of the novel phosphosites identified was thr-66 [19]. Further investigations of this site were conducted based on previous structural studies placing it within eIF2γ’s switch 1 region [32,33]. As eIF2 forms a ternary complex with met-tRNA_i_ and GTP, the switch 1 region of the molecule undergoes a conformational change in order to allow for complexation [34,35]. Our previous report revealed not only phosphorylation of thr-66, but also established PKC as a kinase with the ability to phosphorylate thr-66 *in vitro*. In the current study, we observed that for cells propagated under log-phase growth conditions, thr-66 on eIF2γ is 71%. Given that a majority of thr-66 is phosphorylated for a cancer cell line under optimal conditions, future discernment of the physiological role of this phosphosite should be of paramount importance. Logically, phosphorylation within this region of the molecule may have a significant impact on the protein’s overall structure and consequently its influence on the conformational change experienced upon GTP binding to eIF2γ.

Two other structurally noteworthy phosphorylation sites are ser-55 and thr-56 [36,37]. Both of these sites reside directly within the nucleotide binding pocket of eIF2γ. As eIF2γ represents the core protein of the eIF2 heterotrimer, it binds directly to eIF2α and eIF2β, yet the α and β subunits do not bind to each other [38,39]. Two-thirds of the ternary complex forms upon GTP binding to the heterotrimer, specifically, to eIF2γ. In its inactive form, GDP is bound along with a magnesium molecule in eIF2γ’s binding pocket. Our quantitative phosphoanalysis revealed that the level of phosphorylation is approximately 85% for both ser-55 and thr-56 combined. As with most phosphorylation quantification protocols, phosphosites residing on the same tryptic peptide are difficult to quantify individually [40]. Nonetheless, a level of 85% phosphorylation within the nucleotide binding pocket raises some intriguing possibilities. Phosphorylation in this binding pocket may potentially present itself as a regulatory mechanism eliciting steric strain, which could prevent binding of an additional nucleotide. Such a high level of phosphorylation within this binding pocket most likely influences the interplay of magnesium and GTP binding. The temporal aspects of kinase and/or phosphatase interplay with GTP and magnesium binding necessitate future investigations. The possibility of enzyme-aided phosphorylation diminishes with such a small binding pocket thus raising questions of this event possibly occurring prior to protein folding. Clearly, future studies are needed to determine the effects of such high levels of phosphorylation within an important region of the protein.

In contrast to those phosphosites near the binding pocket and within the switch 1 region, phosphosites associated with the *C*-terminal portion of the protein, ser-412 and thr-413 have levels at 7% while ser-418 and thr-435 are at or near 70%. Thus, from the current study, we can see the dynamics of the phosphorylation landscape unfold. Residues 412 and 413 reside on the outer face of the molecule and despite the fact that they may be more readily available to kinases as compared to residues 55, 56, and 66, (which lie on the interior of the molecule), their levels of phosphorylation are significantly smaller. Future investigations will determine the significance of these phosphorylation stoichiometries, but for now, we can speculate that phosphorylation does not play a major role in the binding to other nucleotides and/or proteins. The same appears not to be true for either ser-418 or thr-435. Again, future studies will determine these residues’ phosphorylation significance, but undoubtedly, different physiological circumstances and pressures exist on the phosphorylated residues of the *C*-terminal of eIF2γ. Such a variation of phosphorylation may imply a structural necessity adopted by the *C*-terminus during the binding processes. It would be interesting to observe phosphorylation differences not only under different growth conditions, but also when one of the four *C*-terminal sites is mutated and how this effect translates to the overall phosphorylation landscape across the *C*-terminal region.

Lastly, thr-109’s phosphorylation level at 30% seems to neither weaken nor strengthen the importance of this phosphosite to the overall physiology of eIF2. However, as stated in our previous report, this residue does reside near a zinc binding domain. The electrostatics involved with the zinc cation and the negative charge inherent in a phosphate moiety could be responsible for an interaction that may determine the proper positioning of the zinc ion. Proteins containing zinc binding motifs have been well documented in their binding affinity for nucleotides. As eIF2 binds to met-tRNA_i_ and is part of the ternary complex that shuttles this polynucleotide to the awaiting mRNA during protein translation initiation, the interplay of the phosphate on thr-109 and the zinc ion may have a meaningful impact on protein translation.

Considerable more research is required to decipher the significance of eIF2β phosphosite structural regions. Previous targeted investigations into eIF2β have established an interaction of the protein with the kinase CK2 and suggest numerous physiological pressures affecting the dynamics of eIF2β phosphorylation warranting further investigation [41]. Nonetheless, our report represents the first measurements of the levels of quantification of these phosphosites. Aside from ser-51 on eIF2α, the phosphosites of eIF2β and eIF2γ are not only dynamic, but may house important functions necessary for translation initiation [42].

Along with eIF2, eIF3 is a necessary initiation factor as it binds to many of the numerous eIFs including eIF4A, eIF1, eIF1A, eIF2, and eIF4G. A critical function of eIF3 is prevention of premature association to the 60S ribosome by binding to the 40S ribosome along with other initiation factors. Although its function and structure have been previously investigated, to date eIF3 has neither a definitive crystal structure nor have any of its phosphorylation sites been thoroughly investigated. Recent studies have shown the structure of the tridodecamer as a 5 lobed entity comprised of a core with five protrusions [43]. The exact structure of each of the lobes has yet to be fully determined, but mass spectrometry along with reconstitution of a recombinant tridodecamer has indicated the core subunits, eIF3a, eIF3b, and eIF3c, as being the three largest [10,44,45]. Interestingly, these same three subunits contain 21 of the 29 possible phosphorylation sites for eIF3. All three of the purported core subunits have high levels of phosphorylation. As the quaternary structure of eIF3 may rely heavily on the integrity of its core proteins, a high level of phosphorylation, especially from log phase grown cells, suggests an increasing role of electrostatic interactions that possibly strengthen the integrity of the entire complex. Levels of phosphorylation were not as elevated for the other phosphorylated subunits except for that of eIF3j. However, eIF3j has been known to disassociate from the other 12 subunits of eIF3 thus a high level of phosphorylation for thr-109 may be indicative of an increased affinity to the holoprotein during translation [7,8]. Future investigations will help determine the nature of each of these phosphosites.

The final protein whose phosphorylation was quantified in this study was eIF4G. A classic example of a scaffolding protein, much like the core proteins of eIF3, phosphorylation was present at its highest level for residues residing in known areas of protein binding. Three phosphorylated residues lie within the eIF3 binding region of eIF4G and all three exhibited high levels of phosphorylation. Other residues that also showed high levels of phosphorylation are residues between the eIF3 and eIF4A binding regions. Although future investigations will decipher the role of these specific phosphosites, phosphosite ser-1187 is known in HEK293 cells to function as a substrate for the kinase PKCα and modulates Mnk1 binding [30]. Again, phosphorylated residues implicated in binding appear to have the highest levels of phosphorylation.

## 3. Experimental Section

### 3.1. Purification of eIFs from HeLa Cell Lysate

All proteins for this study, eIF2, eIF3, and eIF4G were enriched from HeLa cell lysate prior to nano-LC-MS/MS according to previously published protocols [19,29]. Briefly, HeLa cell lysate (from approximately 30 L cells) was quickly thawed at 37 °C supplemented in a mixture including 10% glycerin, 1 mM EDTA, 1 mM EGTA, 50 mM NaF, 50 mM beta-glycerol phosphate, 10 mM benzamidine, 1 mM DTT, and 1× protease inhibitor mixture (Roche, Basel, Switzerland). After stirring for 10 min at 4 °C, the mixture was centrifuged at 20,000× *g* for 20 min at 4 °C. To the resulting supernatant, KCl was added to a final concentration of 450 mM followed by centrifugation in a Beckman Ti-45 rotor for 4 h at 4 °C at 45,000 rpm. The middle two-thirds of the supernatant was carefully removed and stirred at 4 °C while saturated ammonium sulfate was added to a final concentration of 40%. After stirring on ice for 1 h, the suspension was centrifuged at 20,000× *g* for 10 min at 4 °C and the pellet (referred to as the A cut) was frozen for future use. To the remaining supernatant, ammonium sulfate was added to a final concentration of 70%. After stirring for 1 h, the mixture was centrifuged at 20,000× *g* for 10 min at 4 °C. The pellet (the B cut) was resuspended in 50 mL of buffer A (20 mM HEPES, pH 7.5, 10% glycerol, 1 mM EDTA, 1 mM EGTA, 50 mM NaF, 50 mM β-glycerol phosphate, 10 mM benzamidine, 1 mM DTT) containing 50 mM KCl and dialyzed in two liters of the same buffer for 2.5 h at 4 °C. Following dialysis, the lysate was passed through a 0.2 µm syringe filter and loaded onto a MonoQ (10/10) column (GE Healthcare, Sunnyvale, CA, USA). The column was eluted with a linear gradient of 100 to 500 mM KCl in buffer A at 2 mL/min with 3 mL fractions collected. The fractions that contained the protein of interest (either eIF2, eIF3, or eIF4G) were then pooled, dialyzed against buffer A containing 100 mM KCl for 2.5 h at 4 °C, and loaded onto a MonoS (10/10) column (GE Healthcare, Sunnyvale, CA, USA). The same gradient as that of the MonoQ column was applied and fractions from the MonoS column were analyzed using SDS-PAGE in a similar fashion. Fractions containing the protein of interest were pooled and loaded onto a hydroxyapatite column made in-house using commercial hydroxyapatite (Calbiochem, San Diego, CA, USA). Elution was performed using a linear gradient from 0% to 100% 0.5 M potassium phosphate buffer at pH 7.5. Fractions were again analyzed via SDS-PAGE in a similar manner to those eluting from either the MonoQ or MonoS columns. Fractions containing the now purified protein were pooled and concentrated using Amicon Ultra filtration devices with a MWCO of 10,000 Da to yield a final concentration between 1 and 2 mg/mL.

### 3.2. Tryptic Digestion of eIFs with Subsequent Tandem Mass Tag (TMT) Labeling

Each eIF protein was digested with trypsin following the same protocol. Approximately 1 µmol of either eIF2, eIF3, or eIF4G was first reduced at 56 °C for 45 min in 5.5 mM DTT final concentration followed by alkylation for one hour in the dark with iodoacetamide (IAA) added to a final concentration of 10 mM. Trypsin was added at a final enzyme:substrate mass ratio of 1:50 and digestion carried out overnight at 37 °C. The reaction was quenched by flash freezing in liquid nitrogen and the digest was lyophilized. Prior to TMT labeling, each lyophilized sample was dissolved into 100 µL of 2% acetonitrile supplemented with 0.1% trifluoroacetic acid. 

All eIF peptides were prepared for TMT labeling following our published protocol [27]. Briefly, each eIF peptide mixture was divided equally; one population was treated with cerium oxide nanopowder (Sigma-Aldrich, St. Louis, MO, USA) to dephosphorylate the phosphopeptides, the other half was left untreated. Subsequent to dephosphorylation, the dephosphorylated samples were labeled with TMT-126. The remaining untreated half was labeled with TMT-127. Samples were then combined after labeling prior to nano-LC-MS/MS analysis.

### 3.3. Nano-LC-MS/MS of eIF Proteins

All samples underwent complete labeling and were combined as outlined in our protocol [19,27] prior to nano-LC-MS/MS analysis using an LTQ-Orbitrap XL (Thermo Fisher, San Jose, CA, USA) mass spectrometer equipped with an ADVANCE ion max source (Michrom Bioresources Inc., Auburn, CA, USA), a Surveyor MS pump (Thermo Fisher, San Jose, CA, USA), and a microautosampler (Thermo Fisher, San Jose, CA, USA). Samples were loaded onto the column for 30 min at 2% solvent B (0.1% (*v*/*v*) formic acid in acetonitrile) with 98% solvent A at a flow rate of 750 nL/min. Peptides were eluted off the column at 750 nL/min using the following gradient: 2%–10% solvent B for 5 min, 10%–35% solvent B for 65 min, 35%–70% solvent B for 5 min, 35%–70% solvent B for 5 min, 70%–90% solvent B for 5 min, 90% solvent B for 5 min, then reversed to 2% solvent B for 10 min. All ions with TMT labels were analyzed via higher energy collision dissociation (HCD), all others with collision-induced dissociation (CID). Settings for the LTQ-Orbitrap XL were set as follows: data-dependent scan of the top 5 most abundant ions, minimum signal threshold of 55,000, collision energy set to 35%, resolution set to 30,000, dynamic exclusion set to 60 s, repeat count set to 2, and repeat duration set to 30 s. All RAW data were deposited directly into SEQUEST Bioworks 3.3.1 (Thermo Fisher, San Jose, CA, USA) and manually validated. Searches were conducted against an in-house developed database consisting of 584 proteins containing the sequences of all known mammalian eukaryotic initiation factors as well as their reversed sequences. Common contaminants including human keratins, porcine trypsin, bovine serum albumin, bovine beta-casein, as well as their reversed sequences were also included. We performed searches with tryptic specificity and allowed for three missed cleavages at a tolerance of 20 ppm in MS mode and 0.2 Da in MS^2^ mode. Possible structure modifications included for consideration were *N*-terminal and lysine TMT labeling, methionine oxidation, carbamidomethylation of cysteine, and serine, threonine, and tyrosine phosphorylation. Peptides with Xcorr scores greater than 2.5 were considered for further manual validation. All TMT ratios were measured individually and experiments were carried out in triplicate.

## 4. Conclusions

This study represents the first global attempt to elucidate the levels of phosphorylation for three integral protein complexes in the translation initiation pathway (Table 1). Prior studies have revealed the identification of new and novel phosphosites within proteins. In this current investigation, we have provided quantification of phosphosites that may aid in further research of these important sites of regulation. As phosphorylation is a highly dynamic modification itself involving enzymatic addition and removal, levels of phosphorylation may be indicative of a more subtle mechanism of regulation incapable of being observed by identification of novel phosphosites alone. We have concentrated on those sites within this investigation that exhibit high levels of phosphorylation; however, those that display lower levels should not be ignored as gain or absence of function at those residues may be less sensitive to the absolute level of their phosphorylation. Phosphorylation levels appear to be at their highest when the residues are within a region known to be involved in binding to other proteins, metal ions, or nucleic acids. Phosphomimetic studies will ultimately unravel the significance of these phosphosites, which may be critical to protein structure and function as well as to overall influence on the translation initiation process.

## Supplementary Files

Supplementary File 1

## References

[1] Hershey J. W., Sonenberg N., Mathews M.B. (2012). Principles of translational control: An overview. Cold Spring Harb. Perspect. Biol..

[2] Hinnebusch A.G., Lorsch J.R. (2012). The mechanism of eukaryotic translation initiation: New insights and challenges. Cold Spring Harb. Perspect. Biol..

[3] Fraser C.S. (2009). The molecular basis of translational control. Prog. Mol. Biol. Transl. Sci..

[4] Valasek L., Nielsen K.H., Zhang F., Fekete C.A., Hinnebusch A.G. (2004). Interactions of eukaryotic translation initiation factor 3 (eIF3) subunit NIP1/c with eIF1 and eIF5 promote preinitiation complex assembly and regulate start codon selection. Mol. Cell. Biol..

[5] Hinnebusch A.G. (2006). eIF3: A versatile scaffold for translation initiation complexes. Trends Biochem. Sci..

[6] Jivotovskaya A.V., Valasek L., Hinnebusch A.G., Nielsen K.H. (2006). Eukaryotic translation initiation factor 3 (eIF3) and eIF2 can promote mRNA binding to 40S subunits independently of eIF4G in yeast. Mol. Cell. Biol..

[7] Fraser C.S., Berry K.E., Hershey J.W., Doudna J.A. (2007). eIF3j is located in the decoding center of the human 40S ribosomal subunit. Mol. Cell.

[8] Fraser C.S., Lee J.Y., Mayeur G.L., Bushell M., Doudna J.A., Hershey J.W. (2004). The j-subunit of human translation initiation factor eIF3 is required for the stable binding of eIF3 and its subcomplexes to 40 S ribosomal subunits *in vitro*. J. Biol. Chem..

[9] Chiu W.L., Wagner S., Herrmannova A., Burela L., Zhang F., Saini A.K., Valasek L., Hinnebusch A.G. (2010). The *C*-terminal region of eukaryotic translation initiation factor 3a (eIF3a) promotes mRNA recruitment, scanning, and, together with eIF3j and the eIF3b RNA recognition motif, selection of AUG start codons. Mol. Cell. Biol..

[10] Sun C., Todorovic A., Querol-Audi J., Bai Y., Villa N., Snyder M., Ashchyan J., Lewis C.S., Hartland A., Gradia S. (2011). Functional reconstitution of human eukaryotic translation initiation factor 3 (eIF3). Proc. Natl. Acad. Sci. USA.

[11] Hilbert M., Kebbel F., Gubaev A., Klostermeier D. (2011). eIF4G stimulates the activity of the DEAD box protein eIF4A by a conformational guidance mechanism. Nucleic Acids Res..

[12] Nielsen K.H., Behrens M.A., He Y., Oliveira C.L., Jensen L.S., Hoffmann S.V., Pedersen J.S., Andersen G.R. (2011). Synergistic activation of eIF4A by eIF4B and eIF4G. Nucleic Acids Res..

[13] Ozes A.R., Feoktistova K., Avanzino B.C., Fraser C.S. (2011). Duplex unwinding and ATPase activities of the DEAD-box helicase eIF4A are coupled by eIF4G and eIF4B. J. Mol. Biol..

[14] Dever T.E. (2002). Gene-specific regulation by general translation factors. Cell.

[15] Dever T.E., Dar A.C., Sicheri F., Mathews M., Sonenberg N., Hershey J.W.B. (2007). 12 The eIF2α kinases. Translational Control in Biology and Medicine.

[16] Kudlicki W., Wettenhall R.E., Kemp B.E., Szyszka R., Kramer G., Hardesty B. (1987). Evidence for a second phosphorylation site on eIF-2 α from rabbit reticulocytes. FEBS Lett..

[17] Proud C.G. (2005). eIF2 and the control of cell physiology. Semin. Cell Dev. Biol..

[18] Ron D., Harding H.P., Mathews M., Sonenberg N., Hershey J.W.B. (2007). 13 eIF2α phosphorylation in cellular stress responses and disease. Translational Control in Biology and Medicine.

[19] Andaya A., Jia W., Sokabe M., Fraser C.S., Hershey J.W., Leary J.A. (2011). Phosphorylation of human eukaryotic initiation factor 2γ: Novel site identification and targeted PKC involvement. J. Proteome Res..

[20] Goodlett D.R., Aebersold R., Watts J.D. (2000). Quantitative *in vitro* kinase reaction as a guide for phosphoprotein analysis by mass spectrometry. Rapid Commun. Mass Spectrom..

[21] Gygi S.P., Rist B., Gerber S.A., Turecek F., Gelb M.H., Aebersold R. (1999). Quantitative analysis of complex protein mixtures using isotope-coded affinity tags. Nat. Biotechnol..

[22] Mann M. (2006). Functional and quantitative proteomics using SILAC. Nat. Rev. Mol. Cell Biol..

[23] Steen H., Jebanathirajah J.A., Springer M., Kirschner M.W. (2005). Stable isotope-free relative and absolute quantitation of protein phosphorylation stoichiometry by MS. Proc. Natl. Acad. Sci. USA.

[24] Stukenberg P.T., Lustig K.D., McGarry T.J., King R.W., Kuang J., Kirschner M.W. (1997). Systematic identification of mitotic phosphoproteins. Curr. Biol..

[25] Thompson A., Schafer J., Kuhn K., Kienle S., Schwarz J., Schmidt G., Neumann T., Johnstone R., Mohammed A.K., Hamon C. (2003). Tandem mass tags: A novel quantification strategy for comparative analysis of complex protein mixtures by MS/MS. Anal. Chem..

[26] Wright C.A., Howles S., Trudgian D.C., Kessler B.M., Reynard J.M., Noble J.G., Hamdy F.C., Turney B.W. (2011). Label-free quantitative proteomics reveals differentially regulated proteins influencing urolithiasis. Mol. Cell. Proteomics.

[27] Jia W., Andaya A., Leary J.A. (2012). Novel mass spectrometric method for phosphorylation quantification using cerium oxide nanoparticles and tandem mass tags. Anal. Chem..

[28] Tan F., Zhang Y., Wang J., Wei J., Cai Y., Qian X. (2008). An efficient method for dephosphorylation of phosphopeptides by cerium oxide. J. Mass Spectrom..

[29] Damoc E., Fraser C.S., Zhou M., Videler H., Mayeur G.L., Hershey J.W., Doudna J.A., Robinson C.V., Leary J.A. (2007). Structural characterization of the human eukaryotic initiation factor 3 protein complex by mass spectrometry. Mol. Cell Proteomics.

[30] Zhang L., Smit-McBride Z., Pan X., Rheinhardt J., Hershey J.W. (2008). An oncogenic role for the phosphorylated h-subunit of human translation initiation factor eIF3. J. Biol. Chem..

[31] LeFebvre A.K., Korneeva N.L., Trutschl M., Cvek U., Duzan R.D., Bradley C.A., Hershey J. W., Rhoads R.E. (2006). Translation initiation factor eIF4G-1 binds to eIF3 through the eIF3e subunit. J. Biol. Chem..

[32] Roll-Mecak A., Alone P., Cao C., Dever T.E., Burley S.K. (2004). X-ray structure of translation initiation factor eIF2γ: Implications for tRNA and eIF2alpha binding. J. Biol. Chem..

[33] Yatime L., Mechulam Y., Blanquet S., Schmitt E. (2006). Structural switch of the gamma subunit in an archaeal aIF2 α γ heterodimer. Structure.

[34] Alone P.V., Cao C., Dever T.E. (2008). Translation initiation factor 2γ mutant alters start codon selection independent of Met-tRNA binding. Mol. Cell. Biol..

[35] Sokabe M., Yao M., Sakai N., Toya S., Tanaka I. (2006). Structure of archaeal translational initiation factor 2βγ-GDP reveals significant conformational change of the β-subunit and switch 1 region. Proc. Natl. Acad. Sci. USA.

[36] Schmitt E., Blanquet S., Mechulam Y. (2002). The large subunit of initiation factor aIF2 is a close structural homologue of elongation factors. EMBO J..

[37] Schmitt E., Naveau M., Mechulam Y. (2010). Eukaryotic and archaeal translation initiation factor 2: A heterotrimeric tRNA carrier. FEBS Lett..

[38] Nika J., Rippel S., Hannig E.M. (2001). Biochemical analysis of the eIF2beta gamma complex reveals a structural function for eIF2alpha in catalyzed nucleotide exchange. J. Biol. Chem..

[39] Westermann P., Nygard O., Bielka H. (1980). The α and γ subunits of initiation factor eIF-2 can be cross-linked to 18S ribosomal RNA within the quaternary initiation complex, eIF-2-Met-tRNAf GDPCP small ribosomal subunit. Nucleic Acids Res..

[40] Wu R., Haas W., Dephoure N., Huttlin E.L., Zhai B., Sowa M.E., Gygi S.P. (2011). A large-scale method to measure absolute protein phosphorylation stoichiometries. Nat. Methods.

[41] Llorens F., Duarri A., Sarro E., Roher N., Plana M., Itarte E. (2010). The *N*-terminal domain of the human eIF2β subunit and the CK2 phosphorylation sites are required for its function. Biochem. J..

[42] Heaney J.D., Michelson M.V., Youngren K.K., Lam M.Y., Nadeau J.H. (2009). Deletion of eIF2β suppresses testicular cancer incidence and causes recessive lethality in agouti-yellow mice. Hum. Mol. Genet..

[43] Siridechadilok B., Fraser C.S., Hall R.J., Doudna J.A., Nogales E. (2005). Structural roles for human translation factor eIF3 in initiation of protein synthesis. Science.

[44] Zhou M., Sandercock A.M., Fraser C.S., Ridlova G., Stephens E., Schenauer M.R., Yokoi-Fong T., Barsky D., Leary J.A., Hershey J.W. (2008). Mass spectrometry reveals modularity and a complete subunit interaction map of the eukaryotic translation factor eIF3. Proc. Natl. Acad. Sci. USA.

[45] Cai Q., Todorovic A., Andaya A., Gao J., Leary J.A., Cate J.H. (2010). Distinct regions of human eIF3 are sufficient for binding to the HCV IRES and the 40S ribosomal subunit. J. Mol. Biol..

