# Supplementary material for: Phosphorylation Stoichiometries of Human Eukaryotic Initiation Factors
Source: Int J Mol Sci. 2014 Jun 27;15(7):11523–38. doi: 10.3390/ijms150711523 (PMC4139797; doi:10.3390/ijms150711523)

Supplementary Information

Figure S1. Spectrum corresponding to the quantification of phosphorylation of Thr-31 for eIF2β. Inset in upper right corner is a zoom in view of the reporter TMT (tandem mass tag) ions.

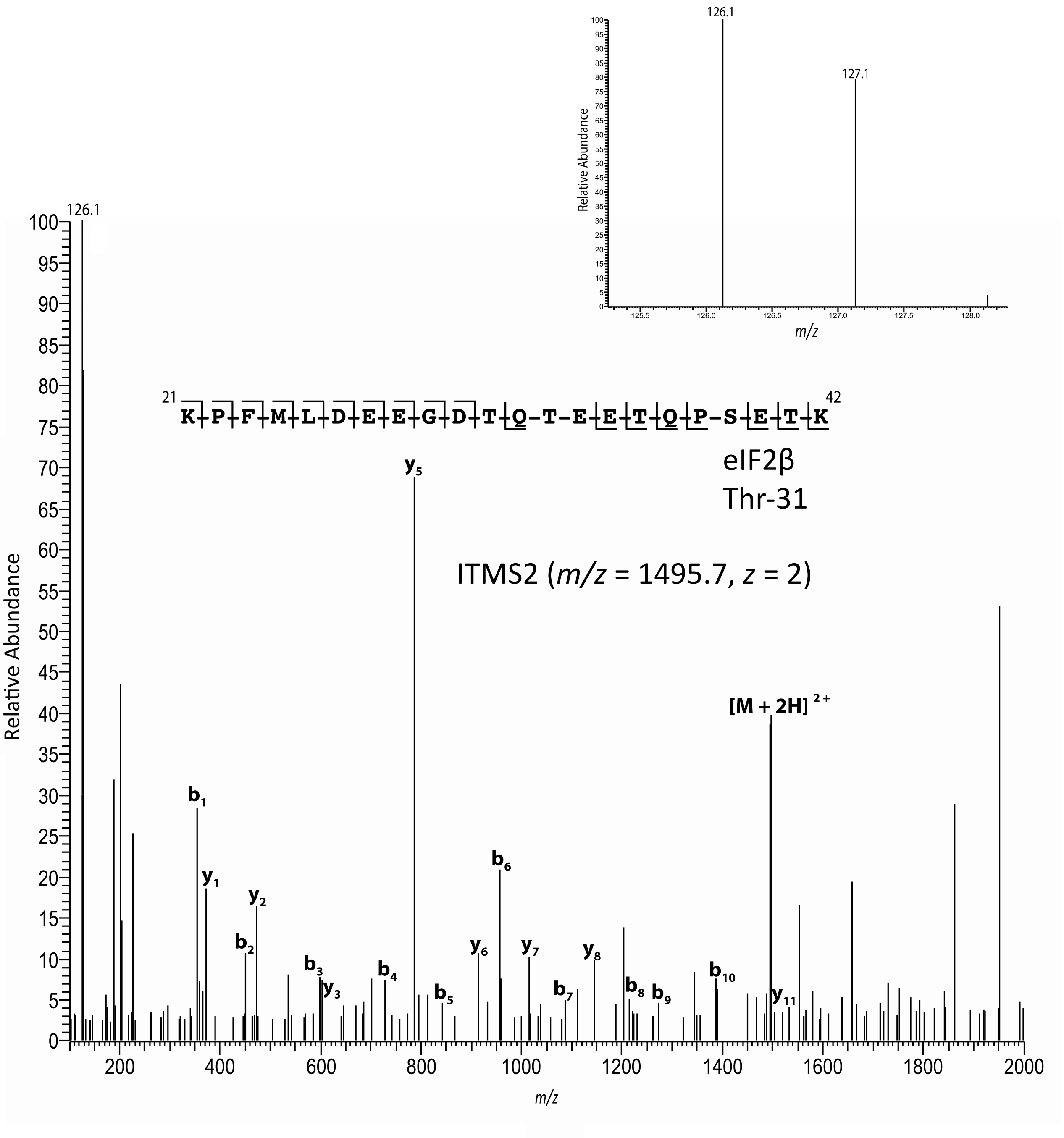

Figure S2. Spectrum corresponding to the quantification of phosphorylation of Ser-67 for eIF2β. Inset in upper right corner is a zoom in view of the reporter TMT ions.

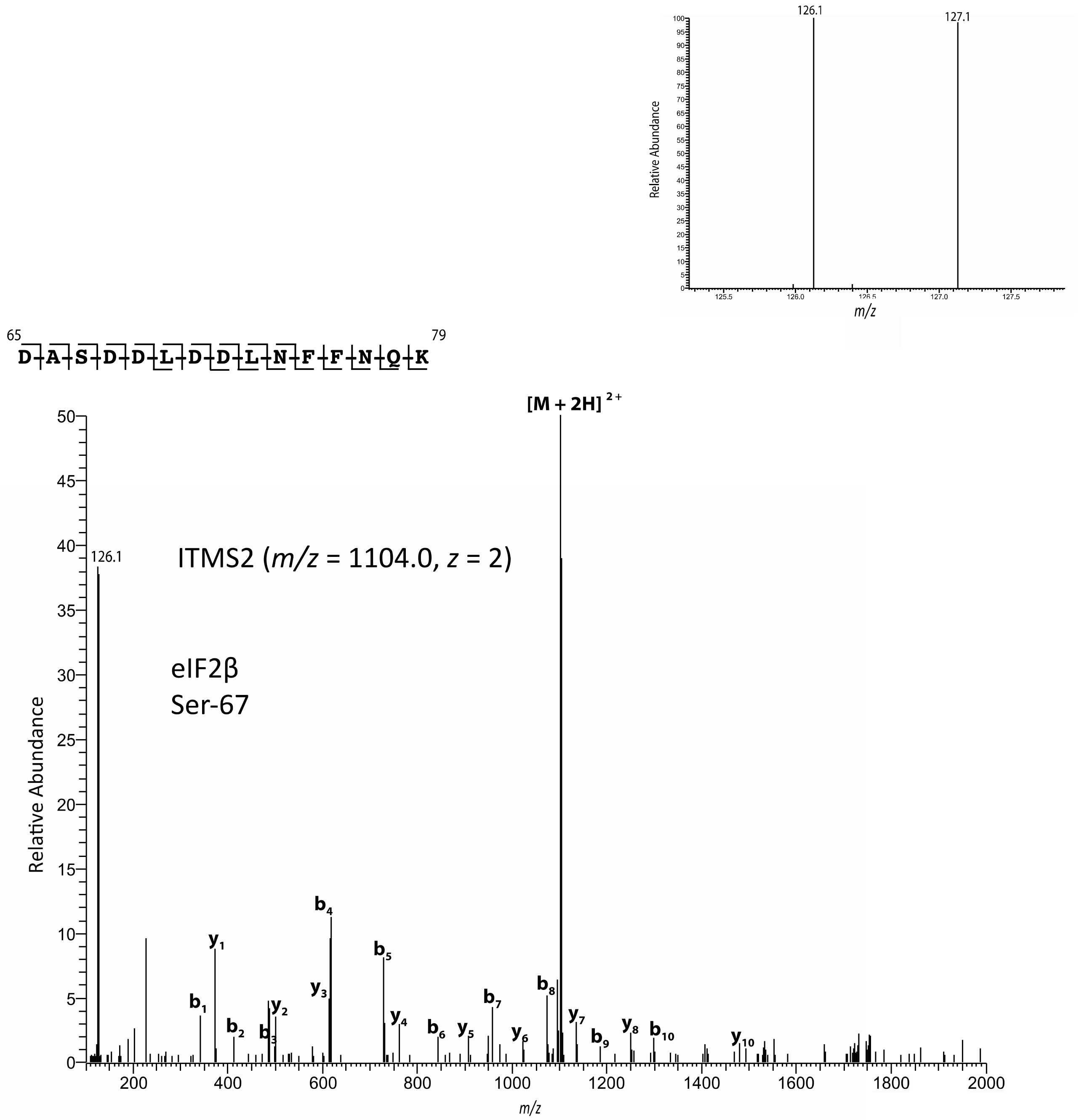

Figure S3. Spectrum corresponding to the quantification of phosphorylation of Ser-105 and Thr-111 for eIF2β. Inset in upper right corner is a zoom in view of the reporter TMT ions.

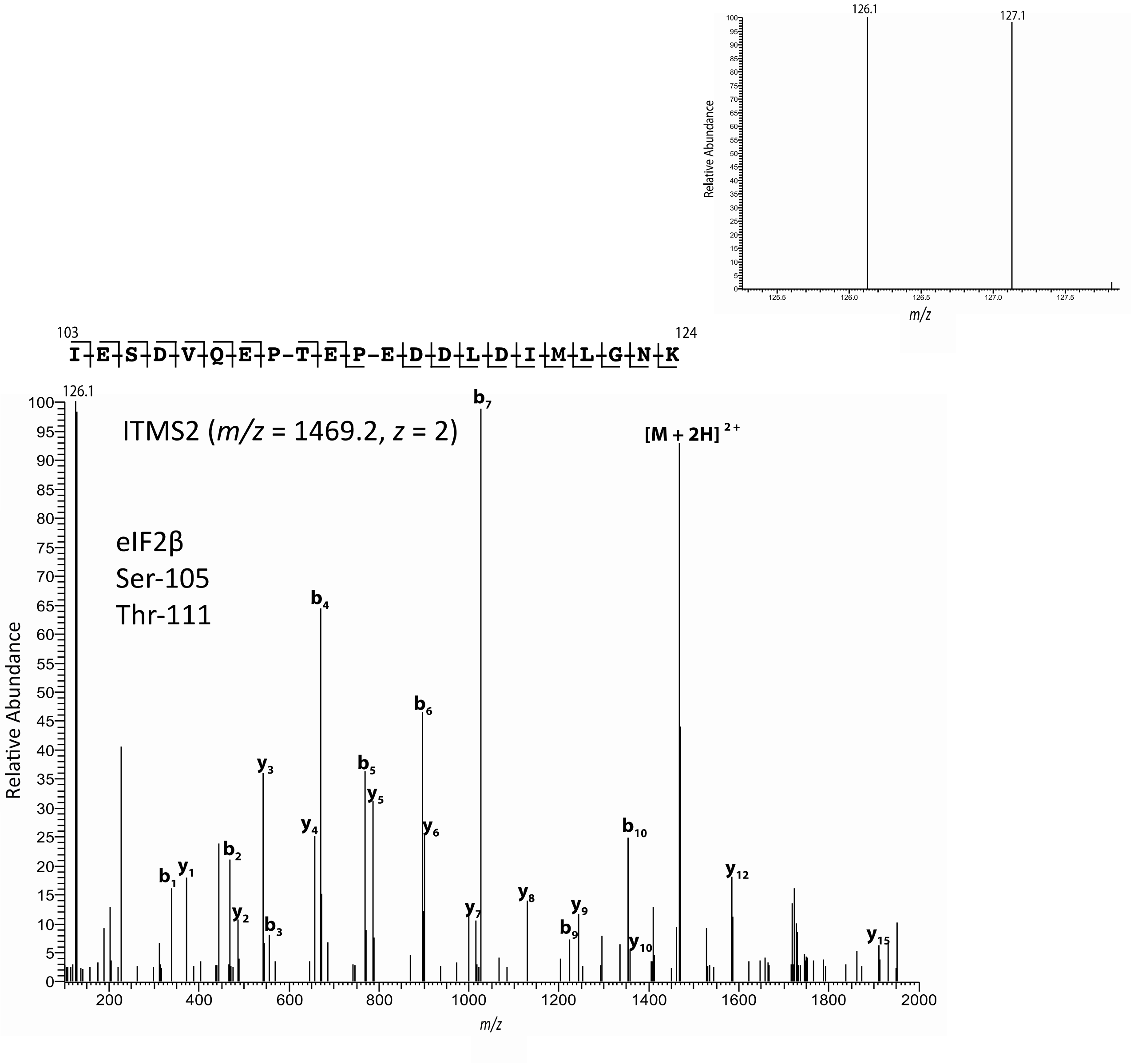

Figure S4. Spectrum corresponding to the quantification of phosphorylation of Ser-158 for eIF2β. Inset in upper right corner is a zoom in view of the reporter TMT ions.

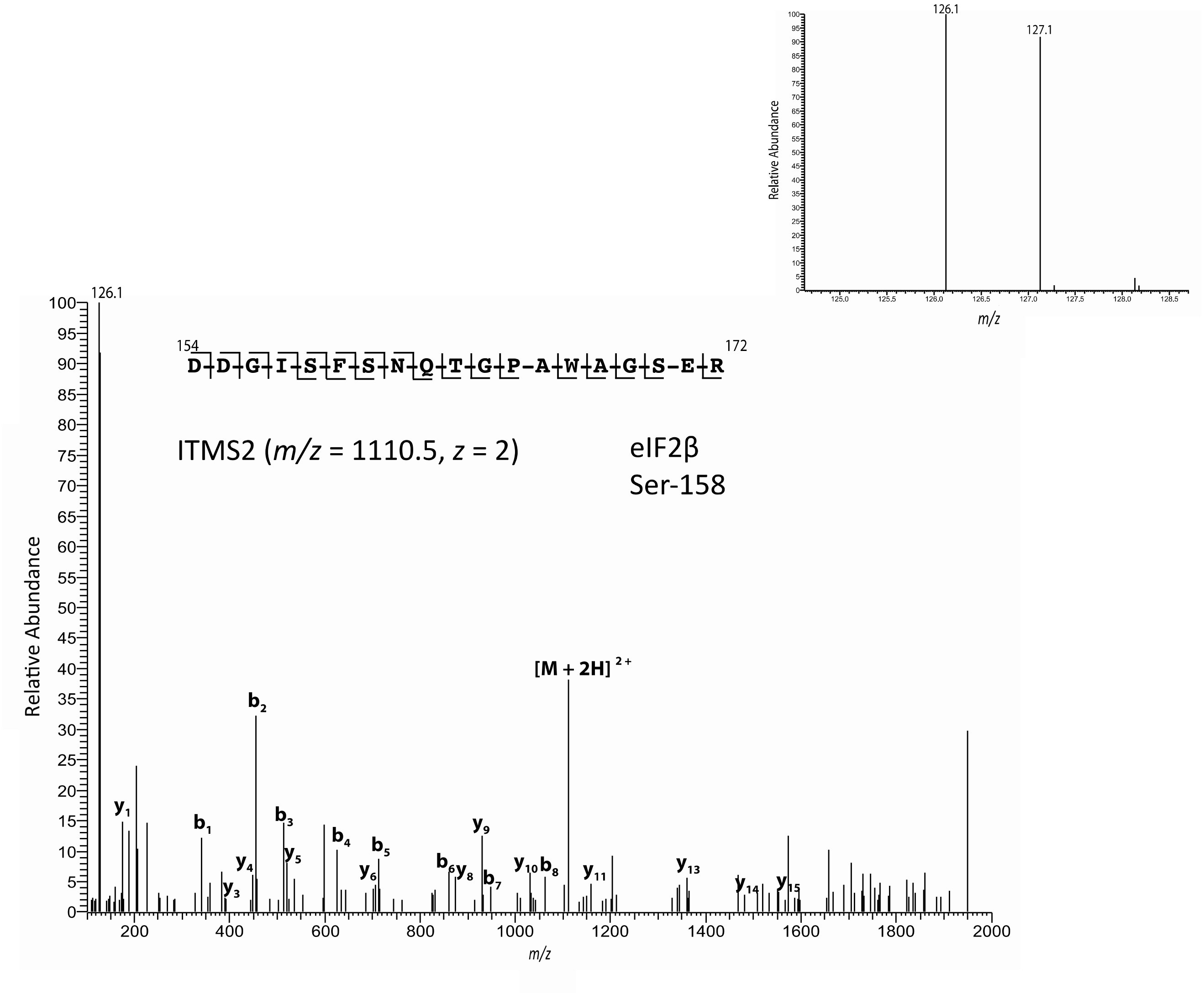

Figure S5. Spectrum corresponding to the quantification of phosphorylation of Ser-55 and Thr-56 for eIF2γ. Inset in upper right corner is a zoom in view of the reporter TMT ions.

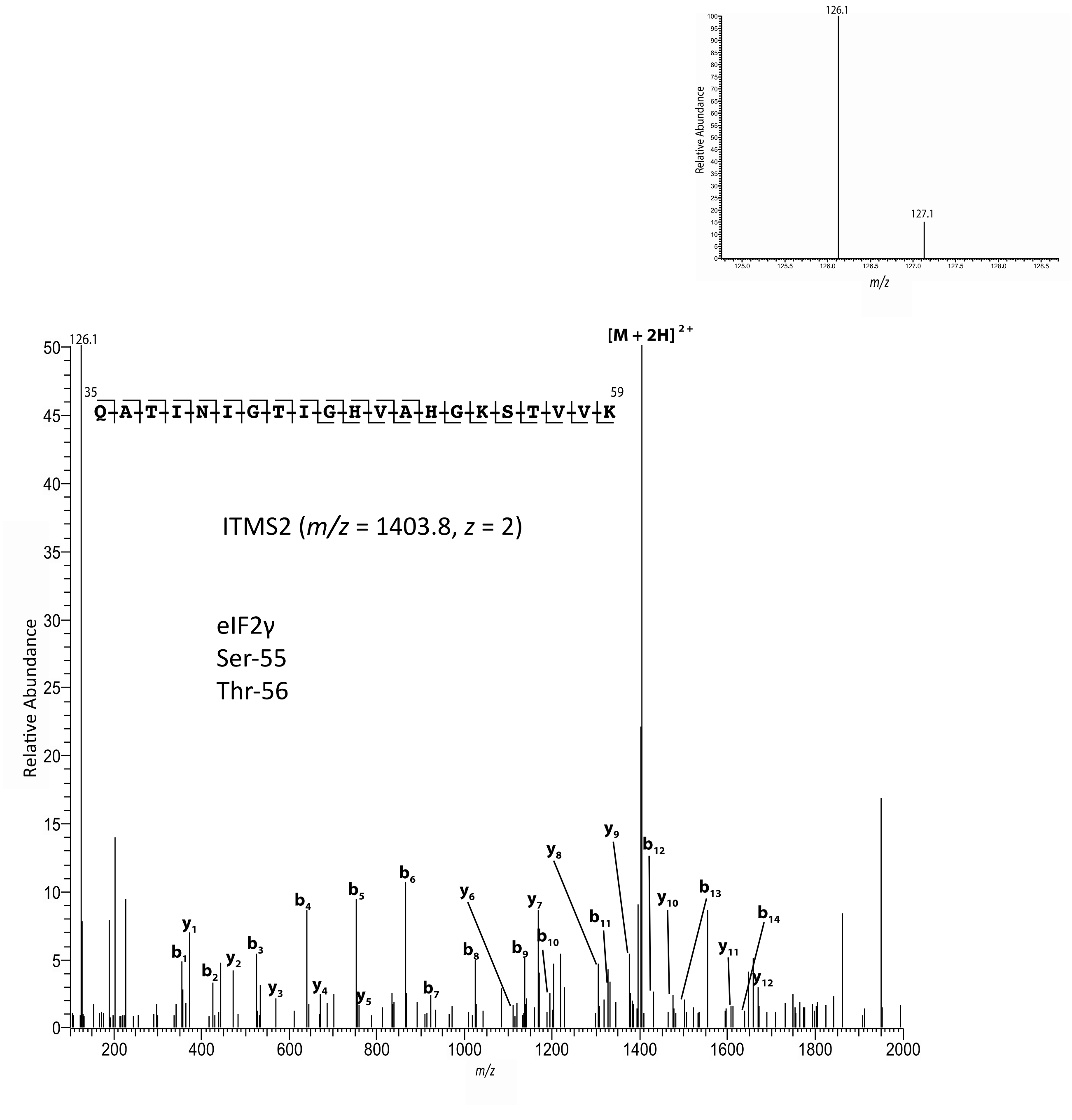

Figure S6. Spectrum corresponding to the quantification of phosphorylation of Thr-109 for eIF2γ. Inset in upper right corner is a zoom in view of the reporter TMT ions.

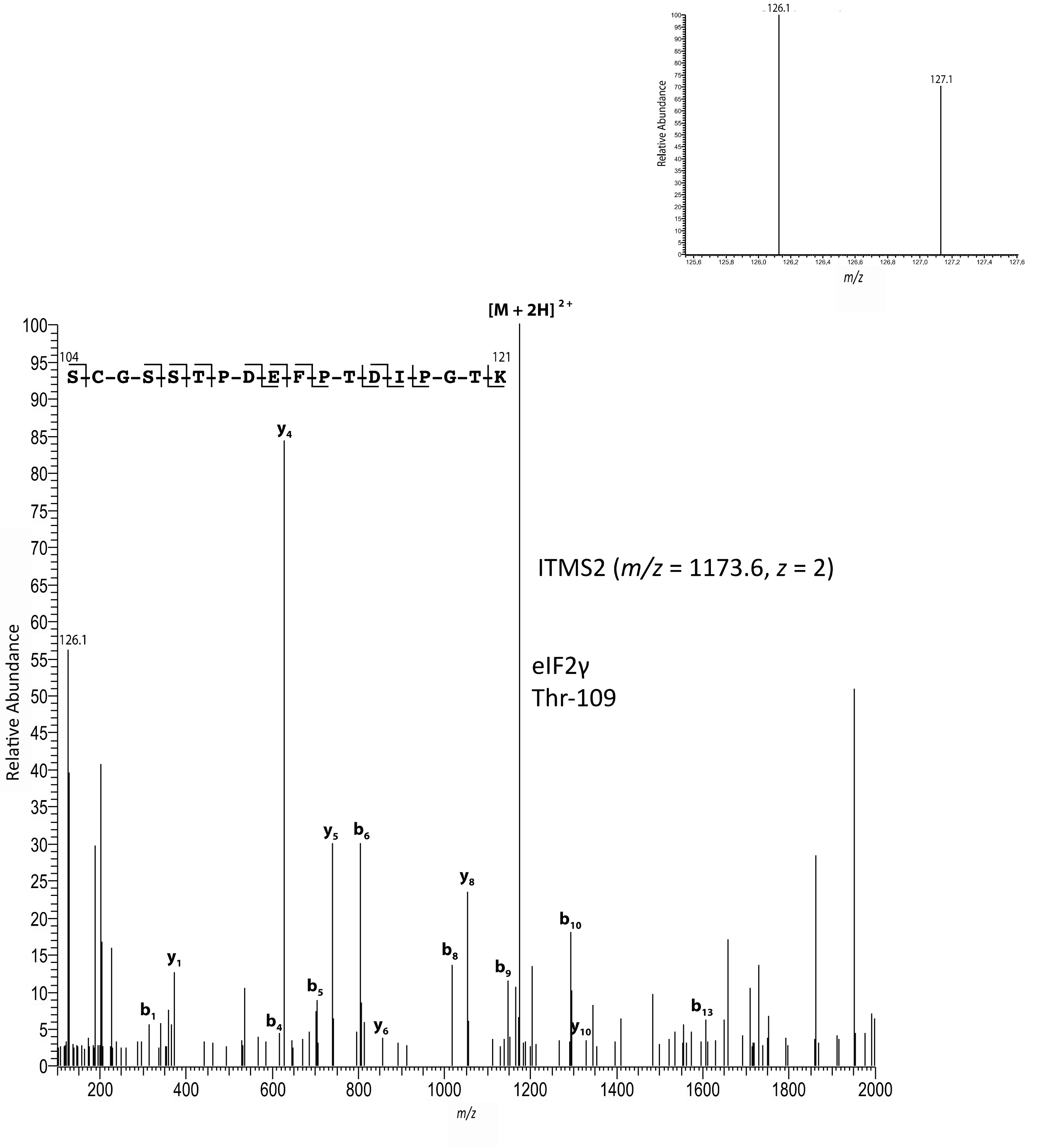

Figure S7. Spectrum corresponding to the quantification of phosphorylation of Ser-412 and Thr-413 for eIF2γ. Inset in upper right corner is a zoom in view of the reporter TMT ions.

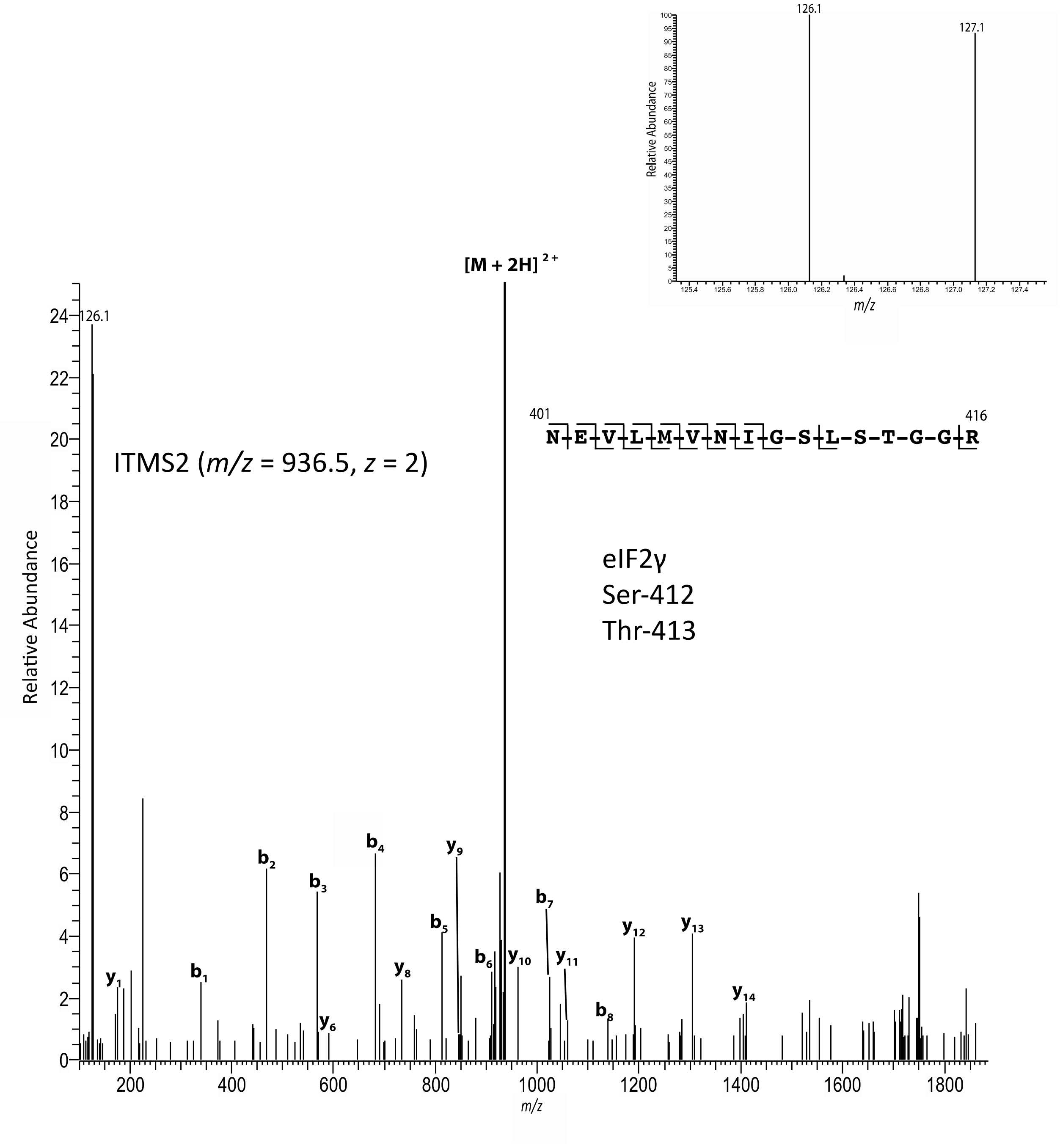

Figure S8. Spectrum corresponding to the quantification of phosphorylation of Ser-418 for eIF2γ. Inset in upper right corner is a zoom in view of the reporter TMT ions.

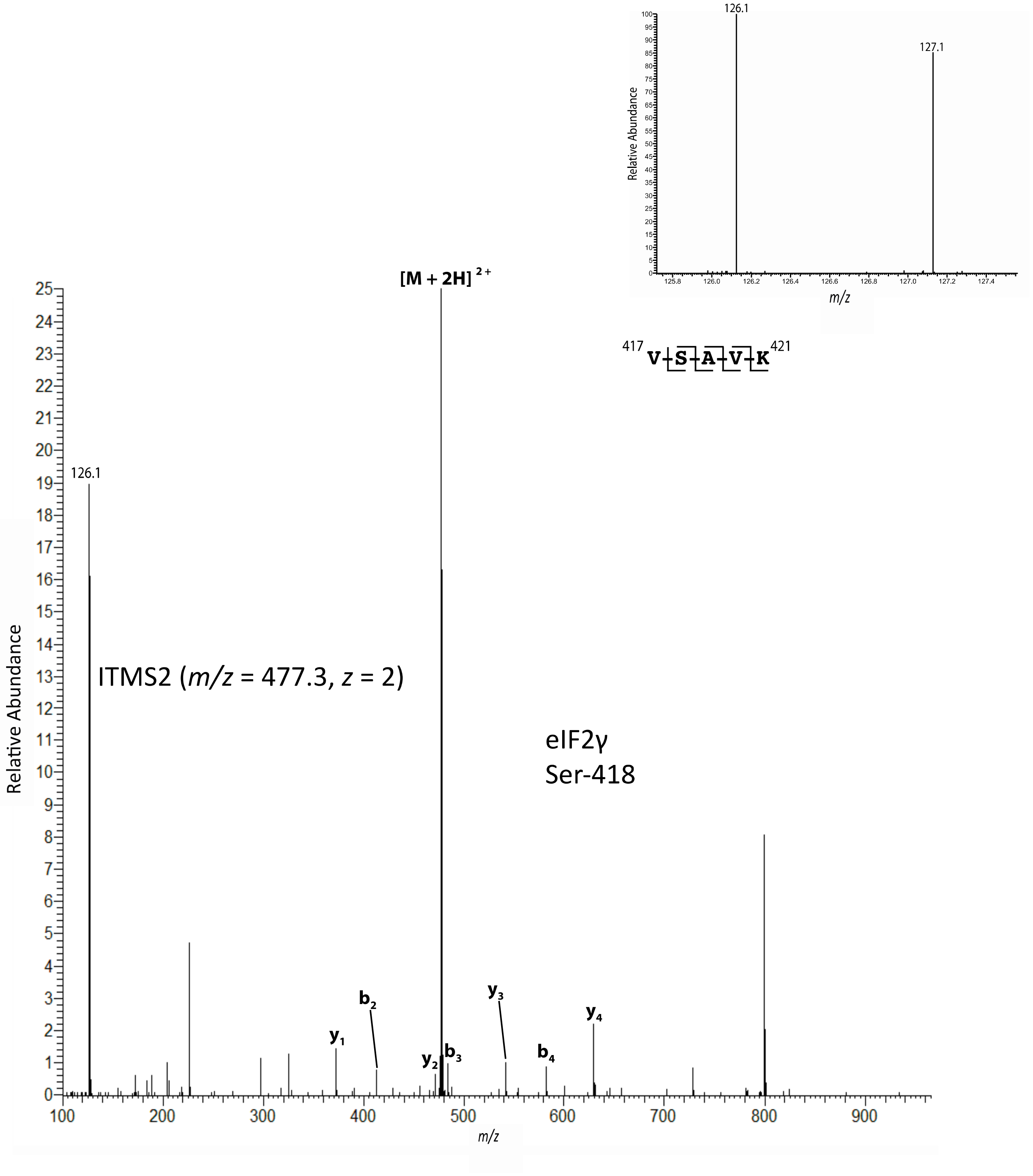

Figure S9. Spectrum corresponding to the quantification of phosphorylation of Thr-435 for eIF2γ. Inset in upper right corner is a zoom in view of the reporter TMT ions.

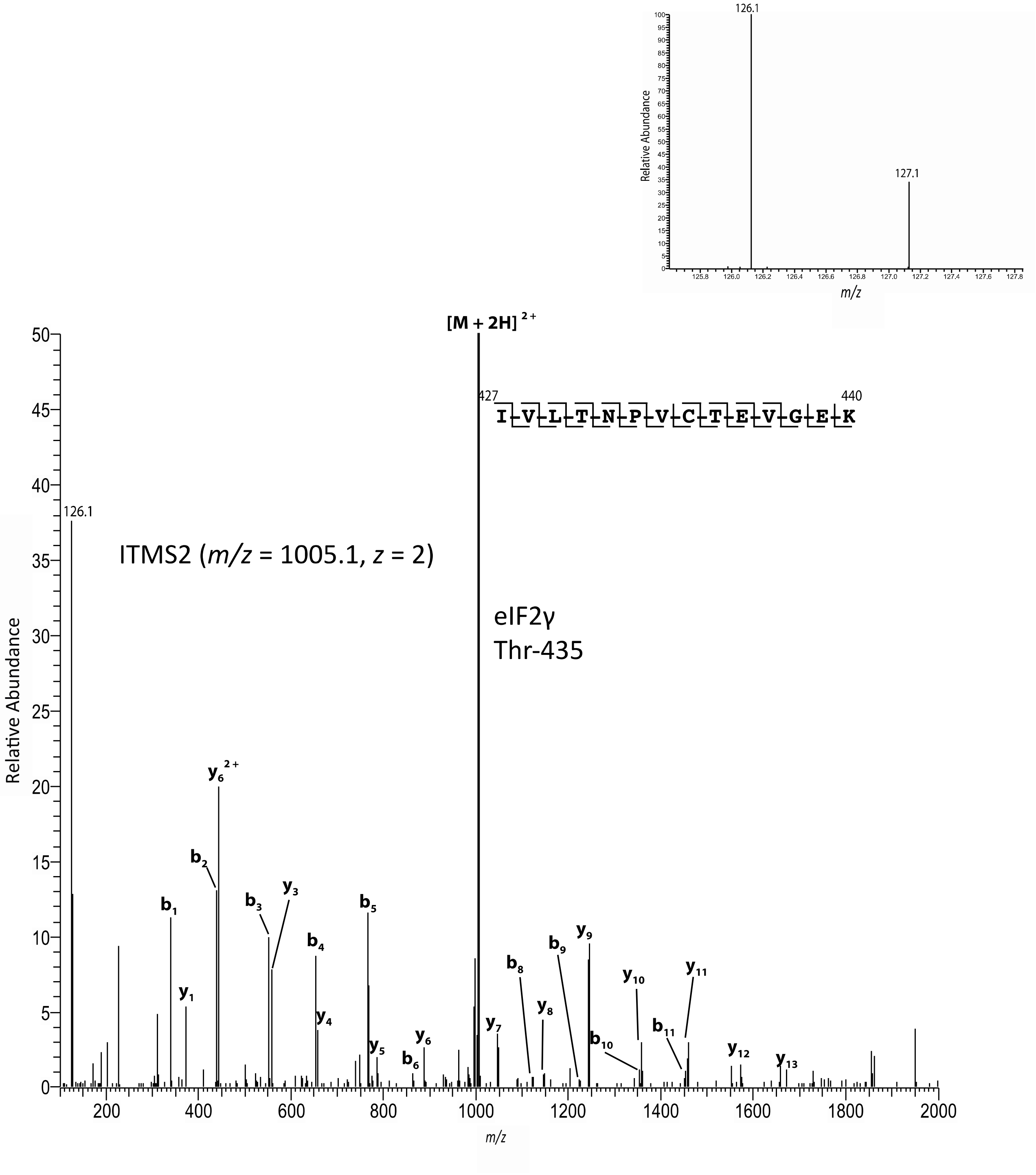

Figure S10. Spectrum corresponding to the quantification of phosphorylation of Ser-881 for eIF3a. Inset in upper right corner is a zoom in view of the reporter TMT ions.

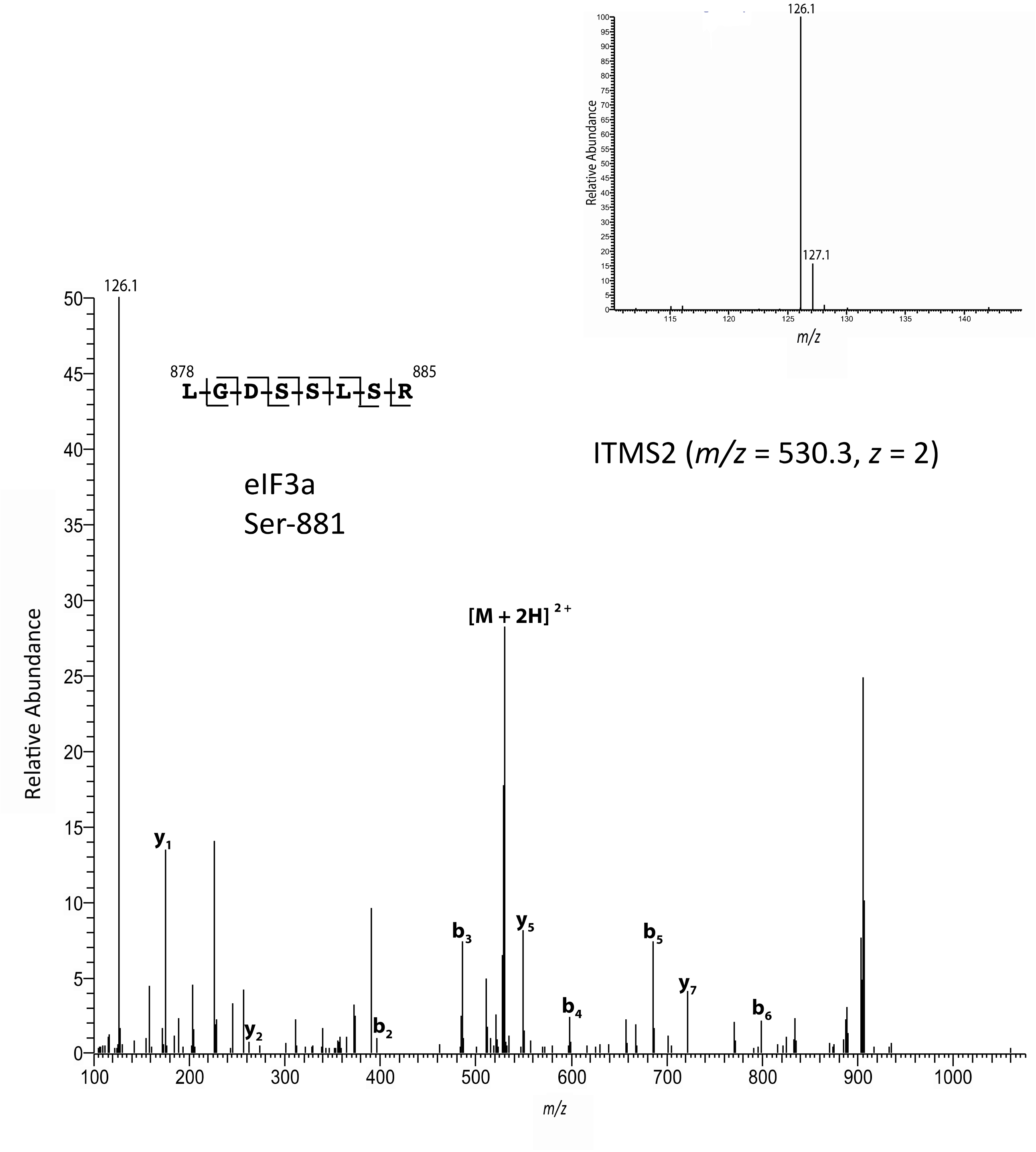

Figure S11. Spectrum corresponding to the quantification of phosphorylation of Ser-1364 for eIF3a. Inset in upper right corner is a zoom in view of the reporter TMT ions.

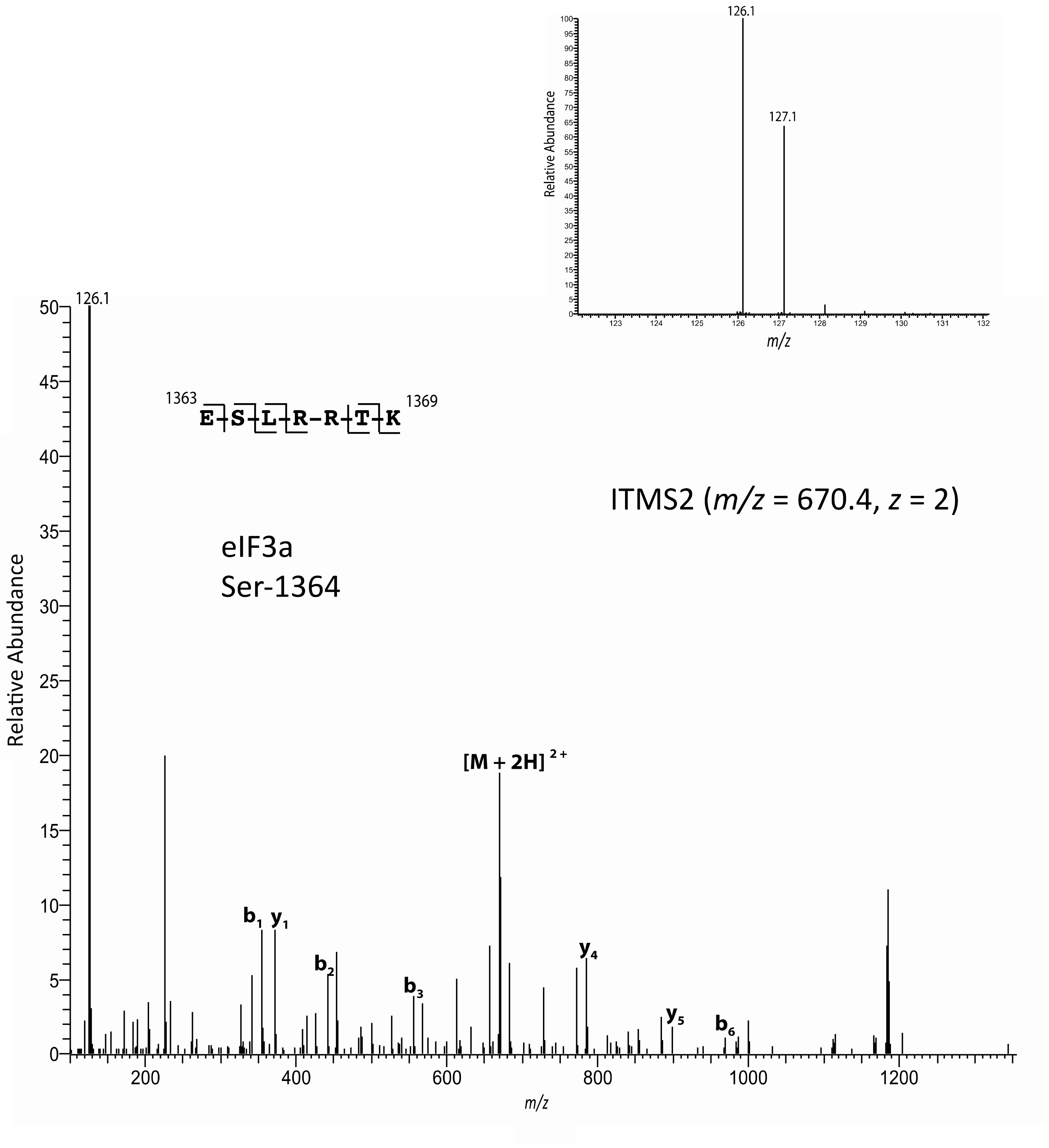

Figure S12. Spectrum corresponding to the quantification of phosphorylation of Ser-83 and Ser-85 for eIF3b. Inset in upper right corner is a zoom in view of the reporter TMT ions.

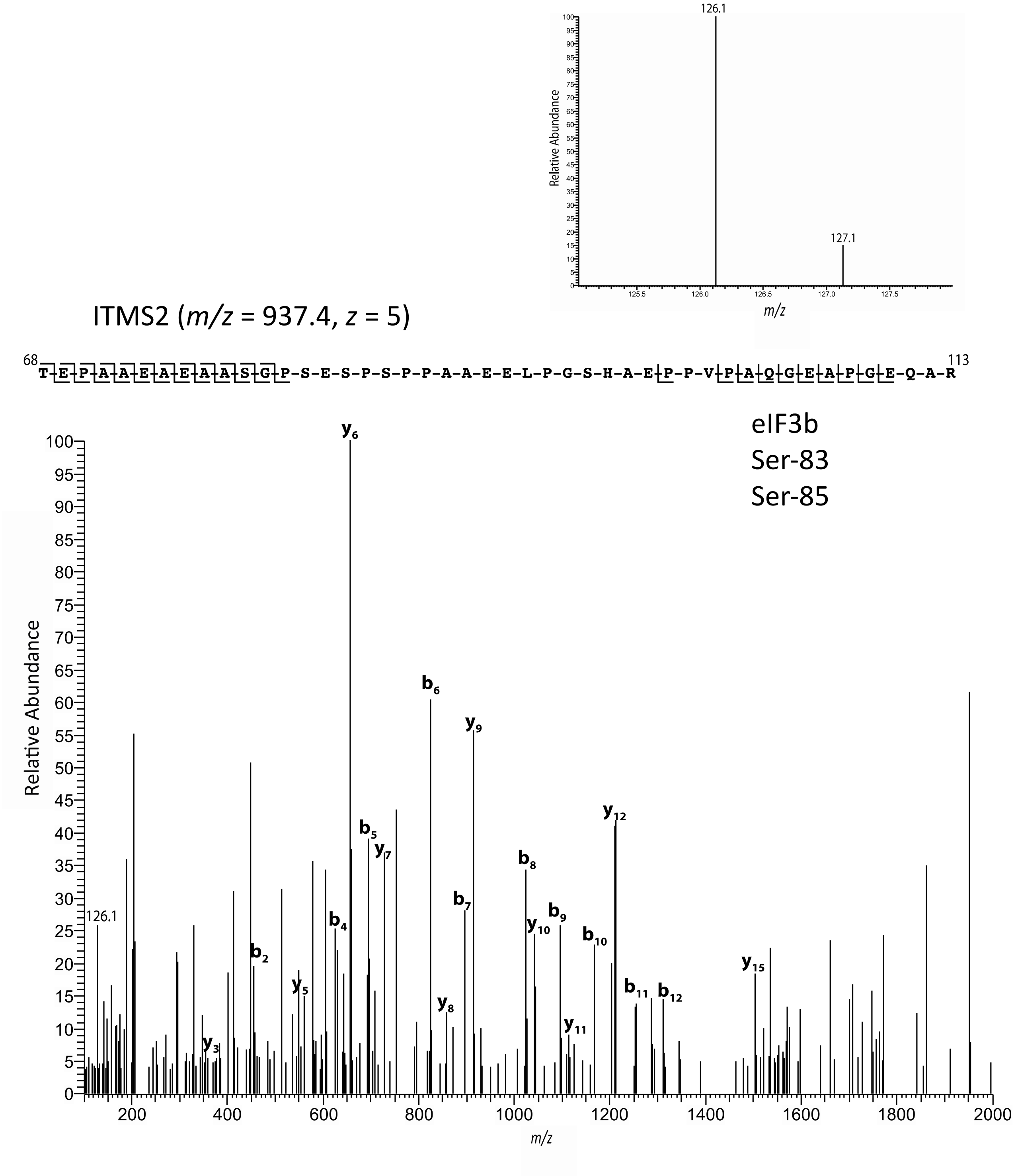

Figure S13. Spectrum corresponding to the quantification of phosphorylation of Ser-125 for eIF3b. Inset in upper right corner is a zoom in view of the reporter TMT ions.

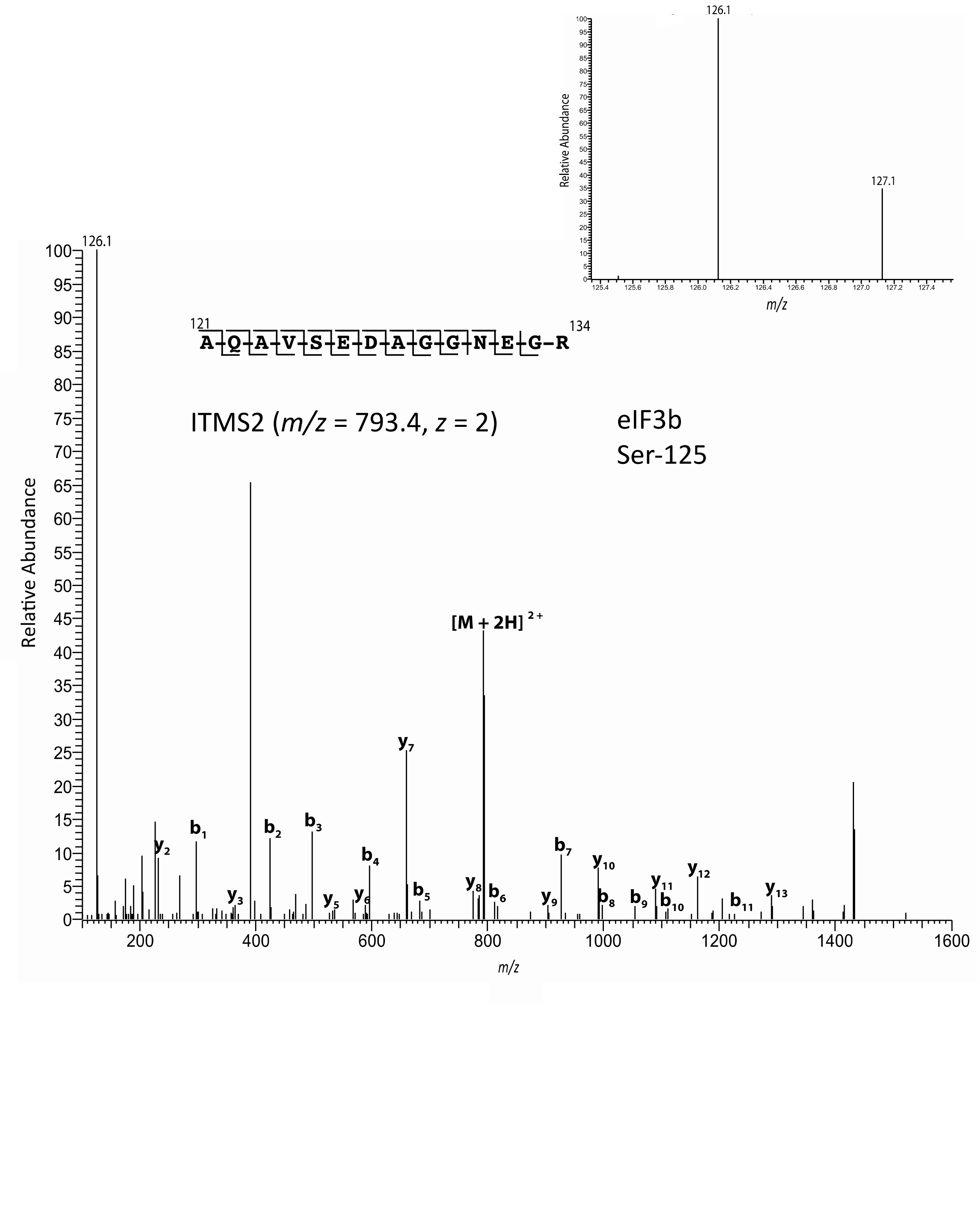

Figure S14. Spectrum corresponding to the quantification of phosphorylation of Thr-524 for eIF3c. Inset in upper right corner is a zoom in view of the reporter TMT ions.

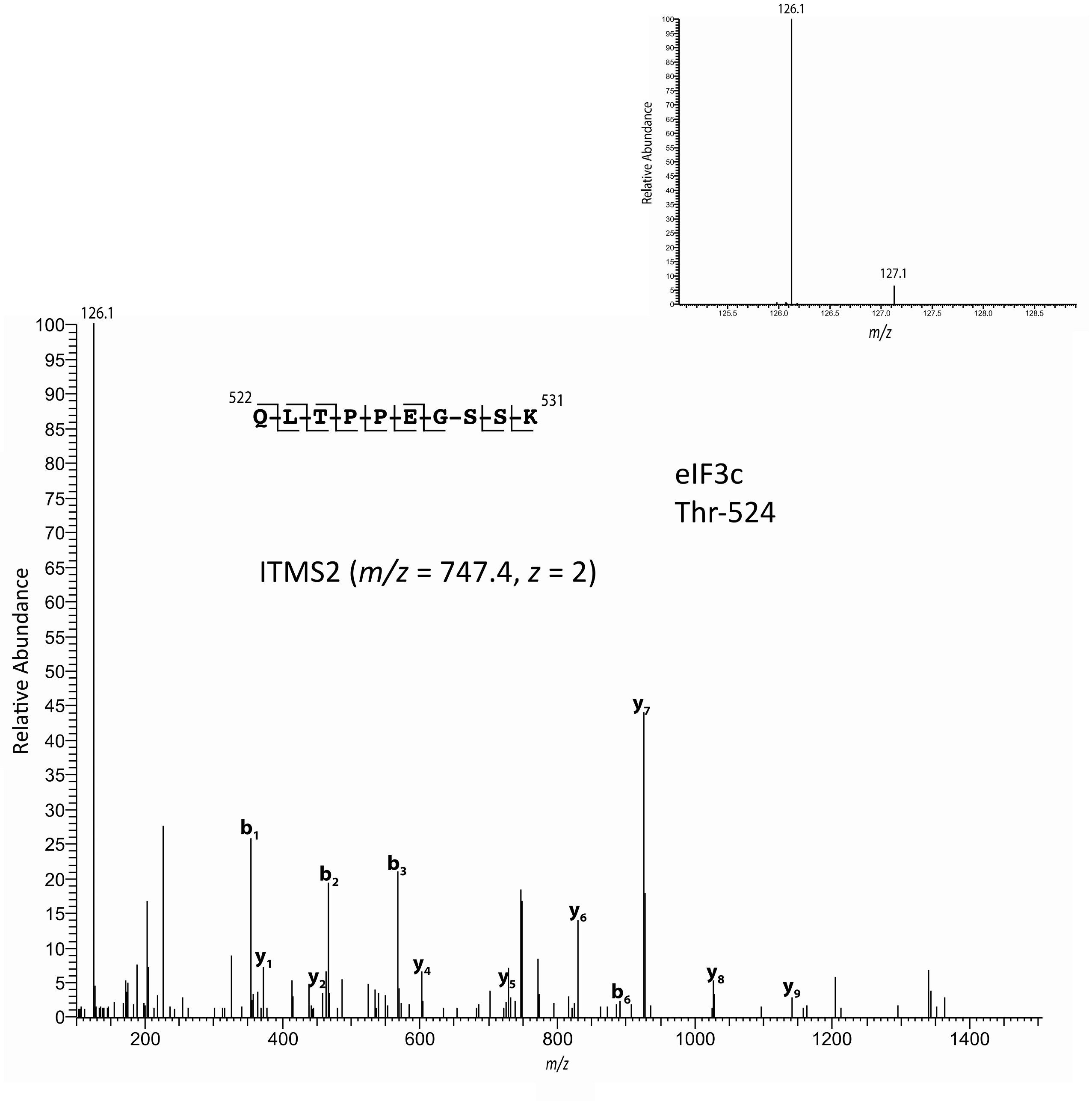

Figure S15. Spectrum corresponding to the quantification of phosphorylation of Thr-41 and Ser-42 for eIF3g. Inset in upper right corner is a zoom in view of the reporter TMT ions.

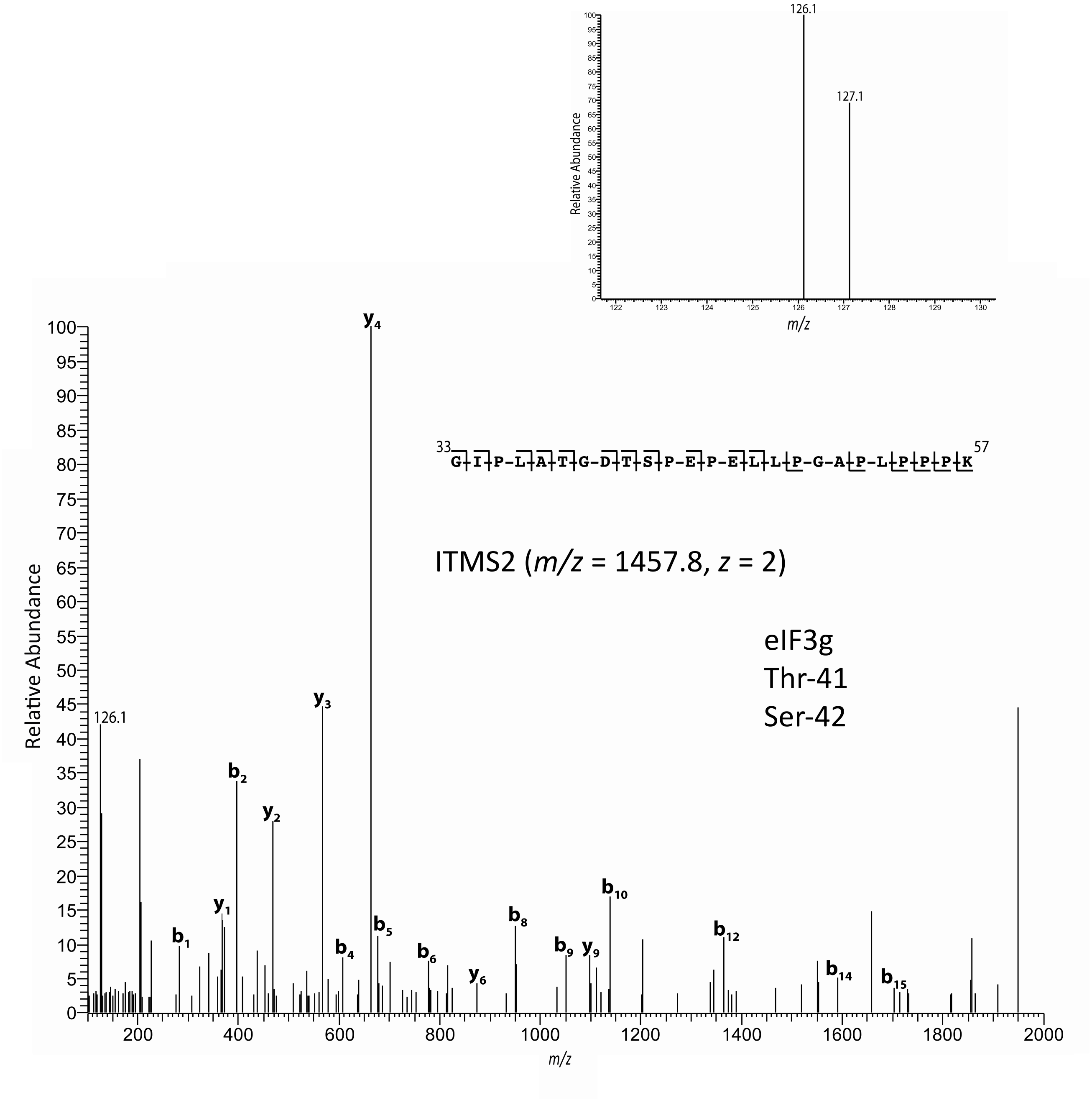

Figure S16. Spectrum corresponding to the quantification of phosphorylation of Ser-183 for eIF3h. Inset in upper right corner is a zoom in view of the reporter TMT ions.

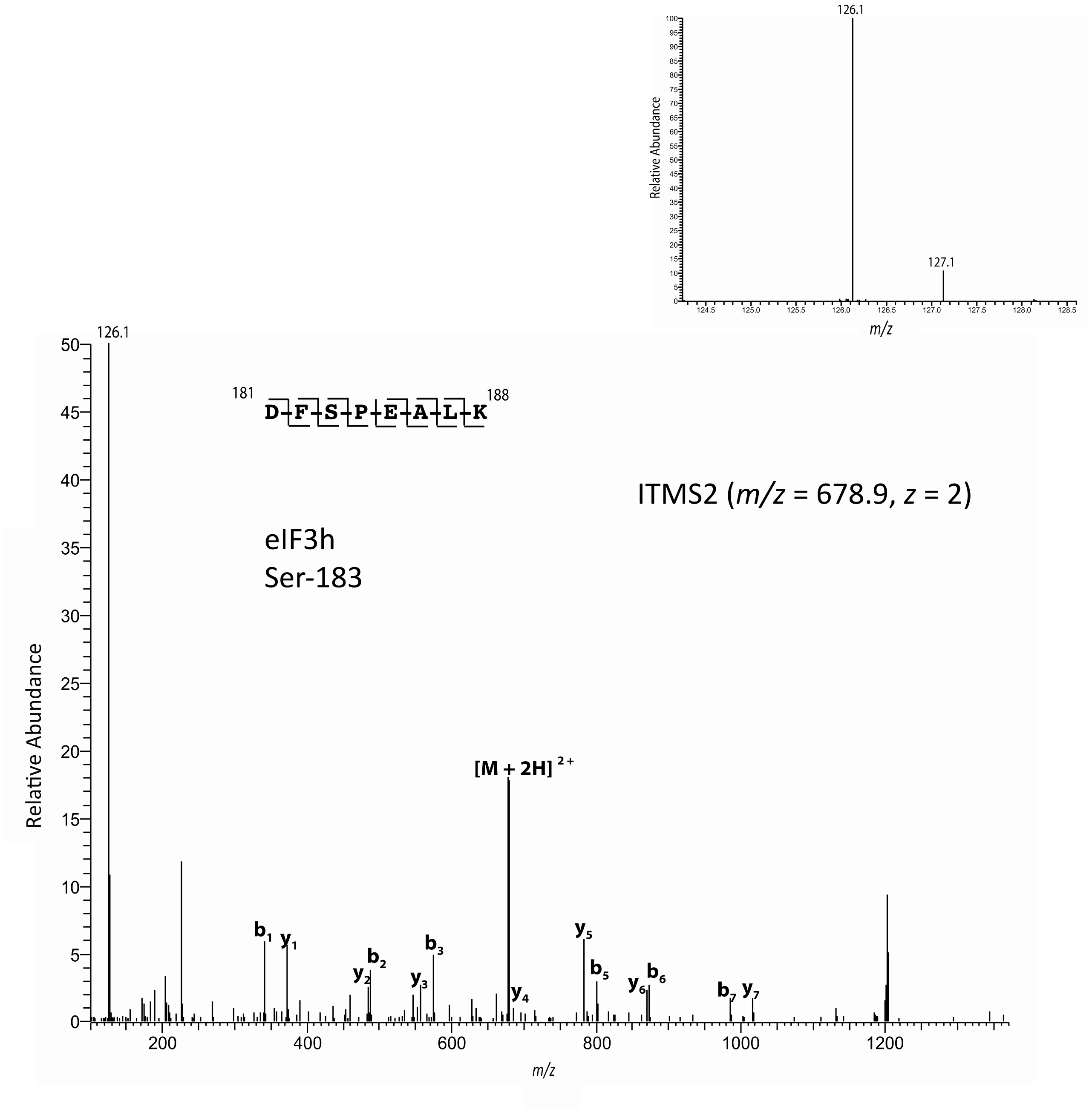

Figure S17. Spectrum corresponding to the quantification of phosphorylation of Thr-109 for eIF3j. Inset in upper right corner is a zoom in view of the reporter TMT ions.

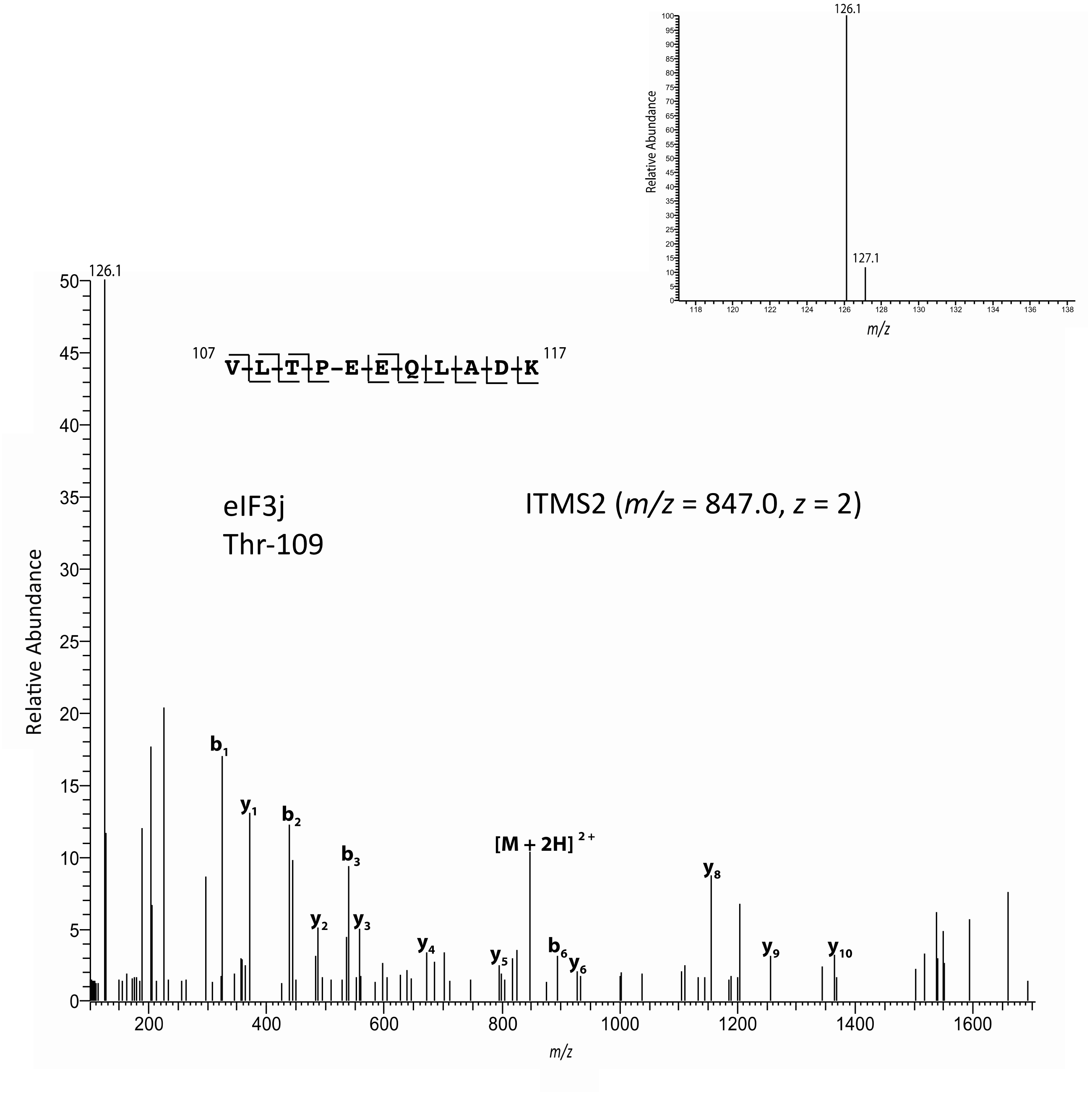

Figure S18. Spectrum corresponding to the quantification of phosphorylation of Thr-647 for eIF4G. Inset in upper right corner is a zoom in view of the reporter TMT ions.

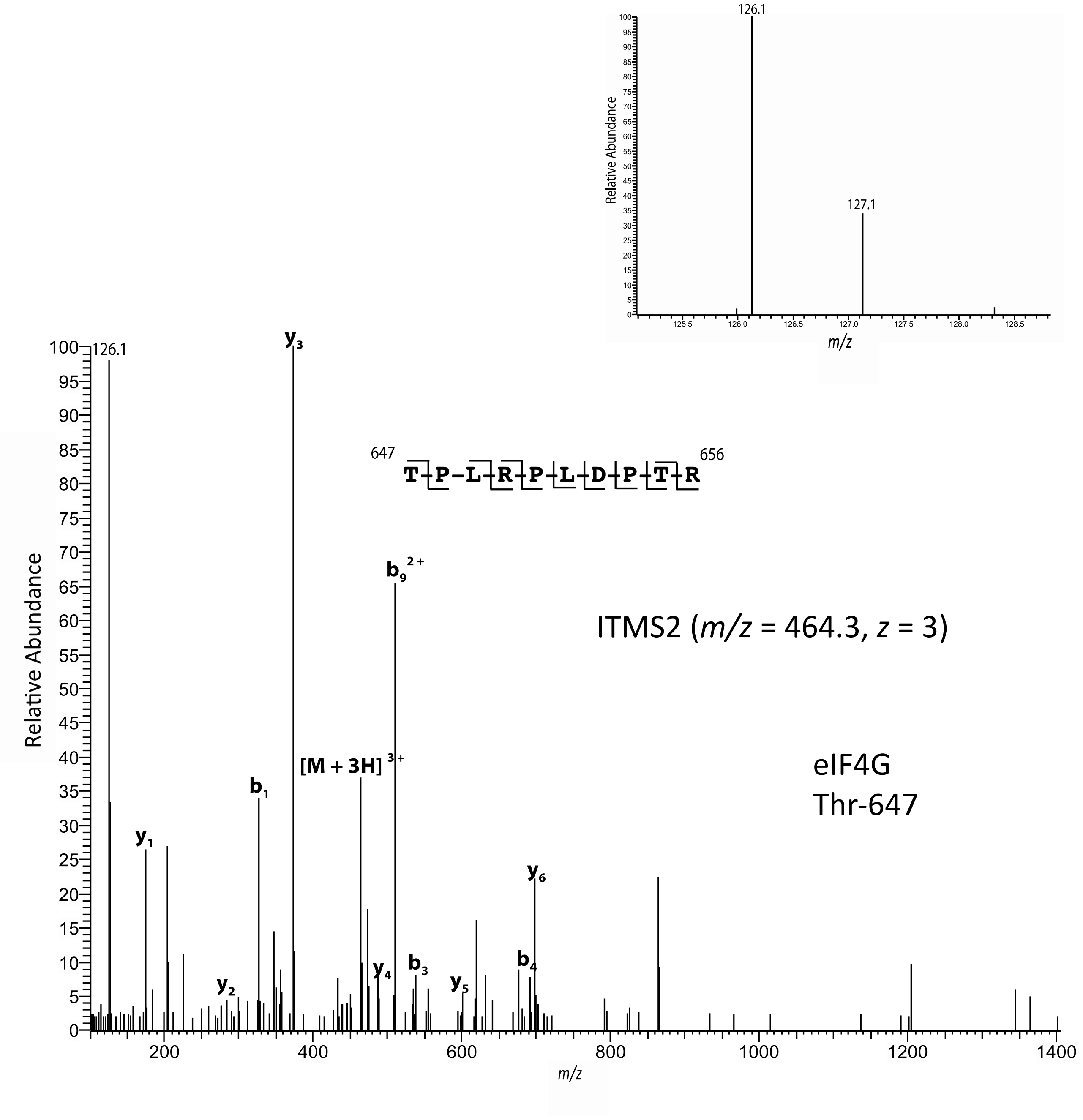

Figure S19. Spectrum corresponding to the quantification of phosphorylation of Ser-1028 for eIF4G. Inset in upper right corner is a zoom in view of the reporter TMT ions.

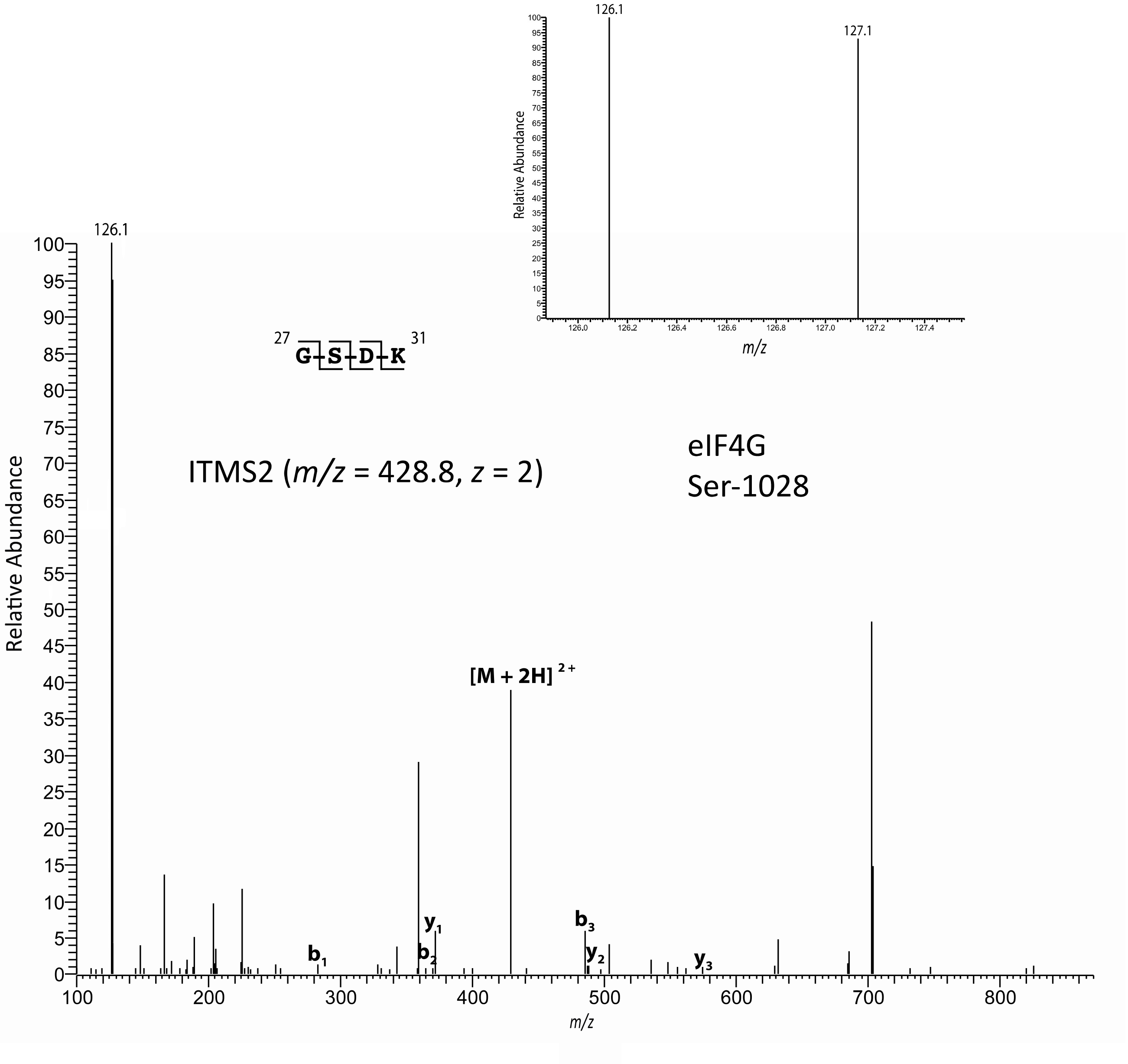

Figure S20. Spectrum corresponding to the quantification of phosphorylation of Ser-1144 and Ser-1147 for eIF4G. Inset in upper right corner is a zoom in view of the reporter TMT ions.

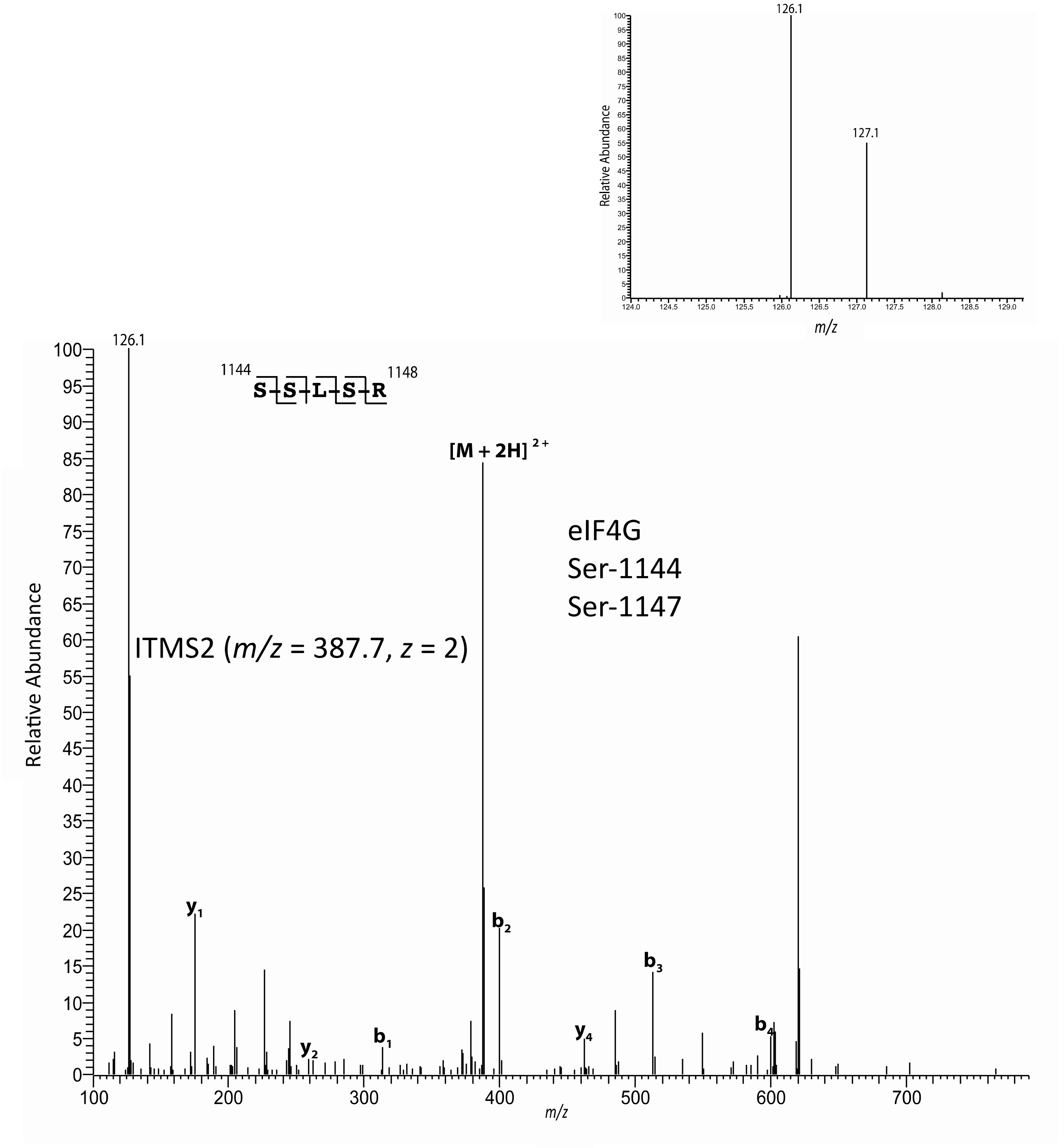

Figure S21. Spectrum corresponding to the quantification of phosphorylation of Ser-1185 and Ser-1187 for eIF4G. Inset in upper right corner is a zoom in view of the reporter TMT ions.

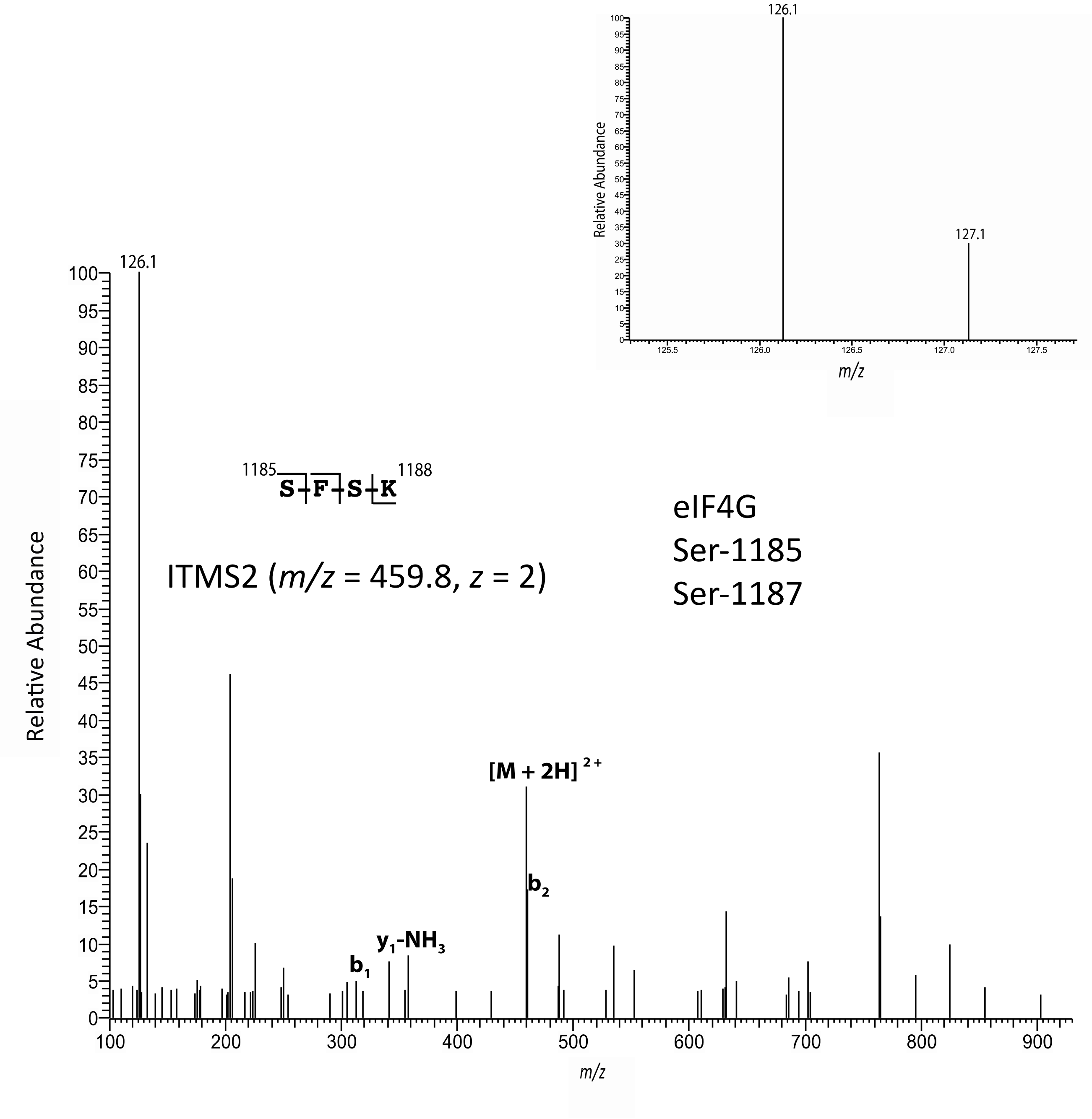

Figure S22. Spectrum corresponding to the quantification of phosphorylation of Ser-1209 and Thr-1211 for eIF4G. Inset in upper right corner is a zoom in view of the reporter TMT ions.

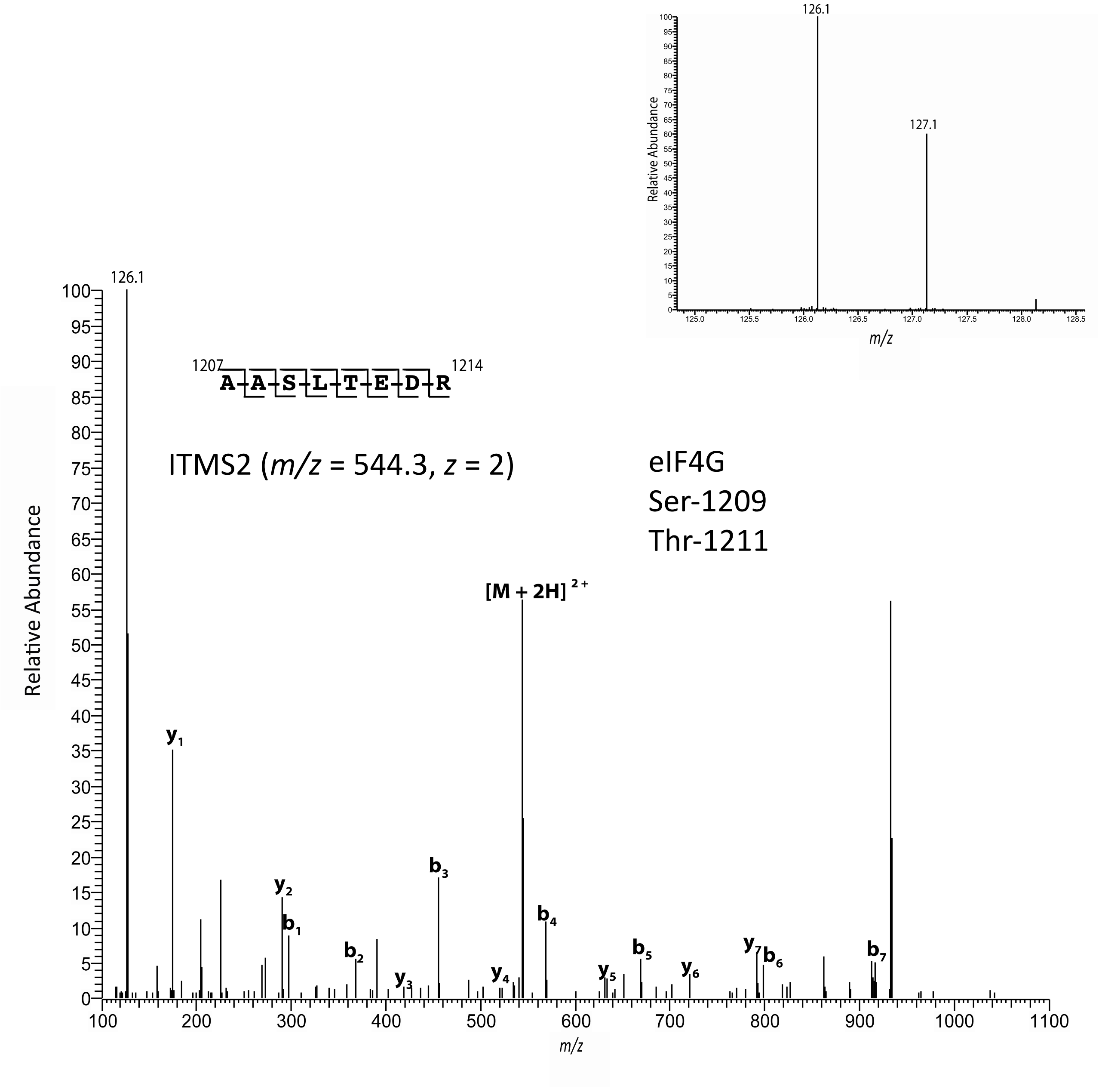

Figure S23. Spectrum corresponding to the quantification of phosphorylation of Ser-1231 for eIF4G. Inset in upper right corner is a zoom in view of the reporter TMT ions.

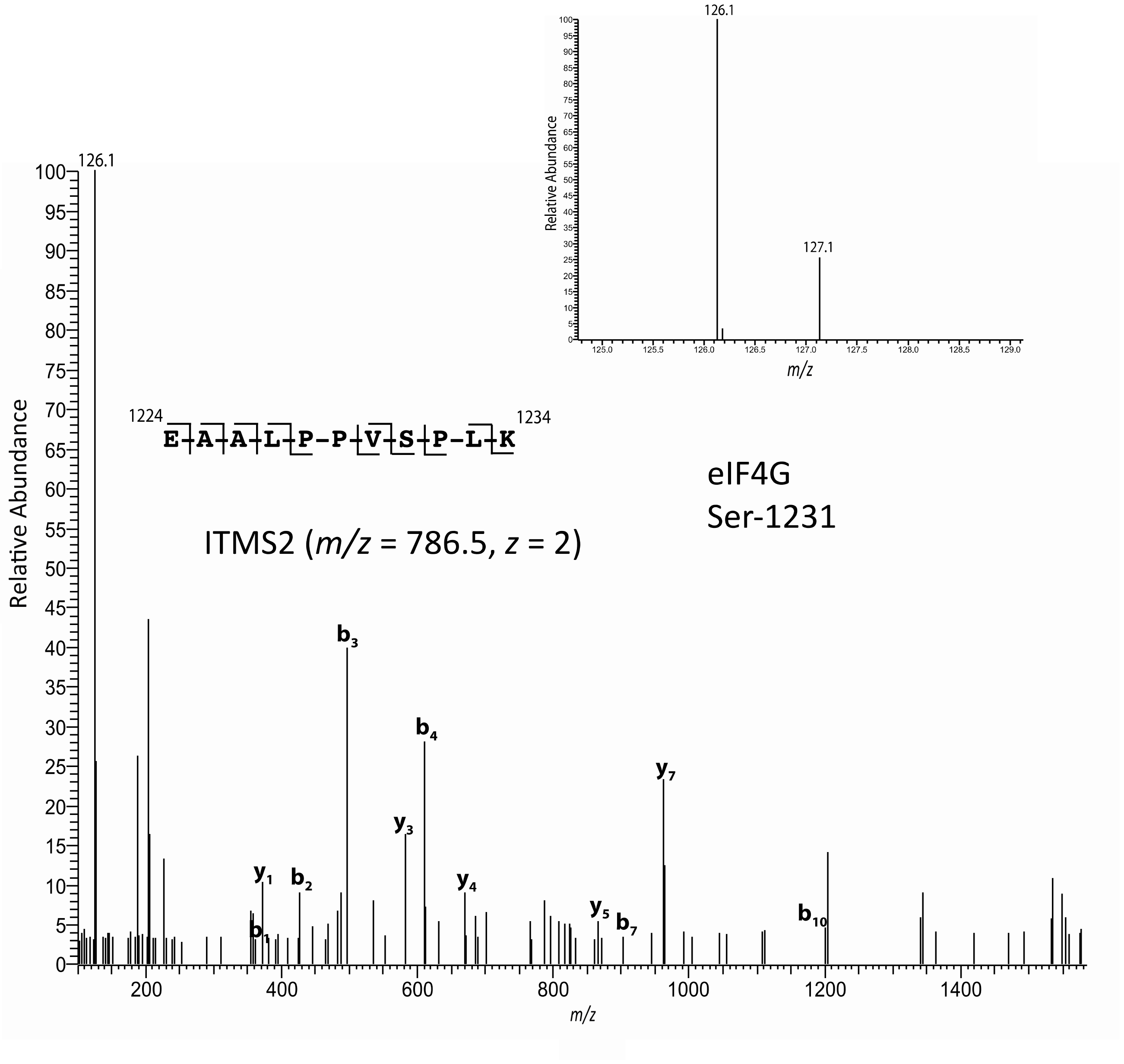

Figure S24. Spectrum corresponding to the quantification of phosphorylation of Thr-1425 and Ser-1430 for eIF4G. Inset in upper right corner is a zoom in view of the reporter TMT ions.

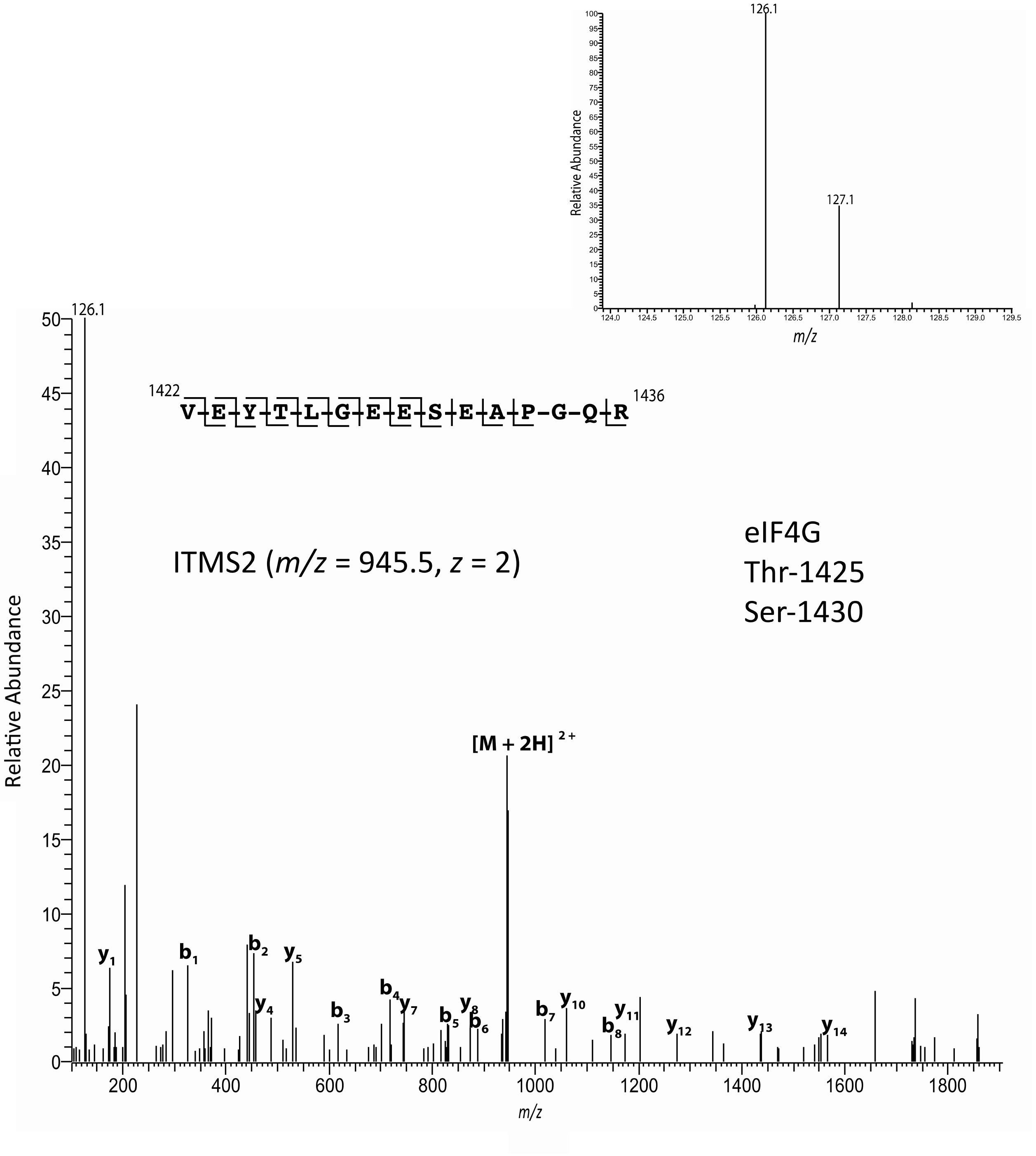

Figure S25. Spectrum corresponding to the quantification of phosphorylation of Ser-1596 for eIF4G. Inset in upper right corner is a zoom in view of the reporter TMT ions.

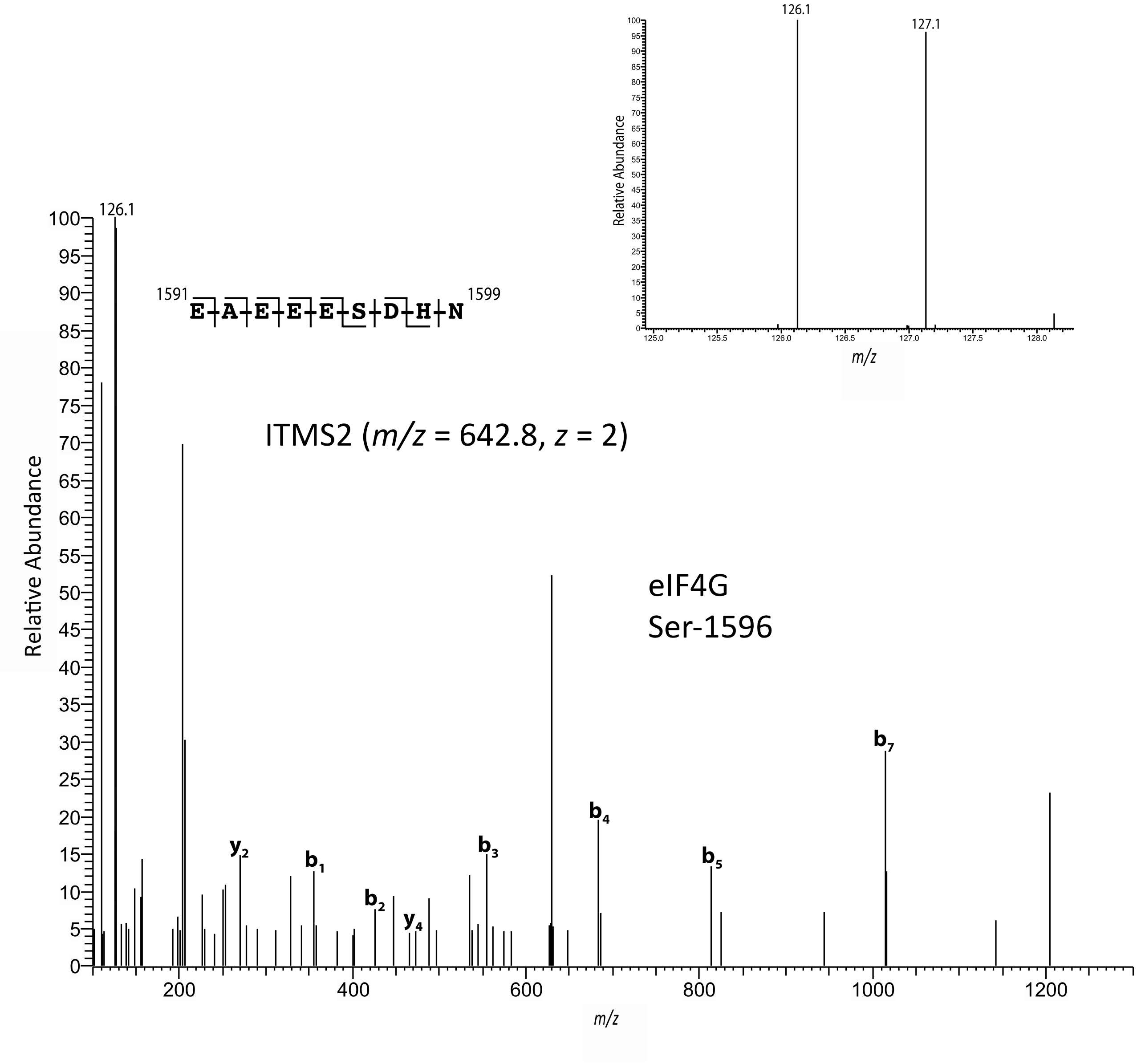

Supplement: Supplementary File 1 [file ijms-15-11523-s001.pdf]
